# Effect of DNA Extraction Methods and Sampling Techniques on the Apparent Structure of Cow and Sheep Rumen Microbial Communities

**DOI:** 10.1371/journal.pone.0074787

**Published:** 2013-09-11

**Authors:** Gemma Henderson, Faith Cox, Sandra Kittelmann, Vahideh Heidarian Miri, Michael Zethof, Samantha J. Noel, Garry C. Waghorn, Peter H. Janssen

**Affiliations:** 1 AgResearch Ltd, Palmerston North, New Zealand; 2 Massey University, Palmerston North, New Zealand; 3 DairyNZ, Hamilton, New Zealand; Uppsala University, Sweden

## Abstract

Molecular microbial ecology techniques are widely used to study the composition of the rumen microbiota and to increase understanding of the roles they play. Therefore, sampling and DNA extraction methods that result in adequate yields of microbial DNA that also accurately represents the microbial community are crucial. Fifteen different methods were used to extract DNA from cow and sheep rumen samples. The DNA yield and quality, and its suitability for downstream PCR amplifications varied considerably, depending on the DNA extraction method used. DNA extracts from nine extraction methods that passed these first quality criteria were evaluated further by quantitative PCR enumeration of microbial marker loci. Absolute microbial numbers, determined on the same rumen samples, differed by more than 100-fold, depending on the DNA extraction method used. The apparent compositions of the archaeal, bacterial, ciliate protozoal, and fungal communities in identical rumen samples were assessed using 454 Titanium pyrosequencing. Significant differences in microbial community composition were observed between extraction methods, for example in the relative abundances of members of the phyla *Bacteroidetes* and *Firmicutes*. Microbial communities in parallel samples collected from cows by oral stomach-tubing or through a rumen fistula, and in liquid and solid rumen digesta fractions, were compared using one of the DNA extraction methods. Community representations were generally similar, regardless of the rumen sampling technique used, but significant differences in the abundances of some microbial taxa such as the *Clostridiales* and the *Methanobrevibacter ruminantium* clade were observed. The apparent microbial community composition differed between rumen sample fractions, and *Prevotellaceae* were most abundant in the liquid fraction. DNA extraction methods that involved phenol-chloroform extraction and mechanical lysis steps tended to be more comparable. However, comparison of data from studies in which different sampling techniques, different rumen sample fractions or different DNA extraction methods were used should be avoided.

## Introduction

Ruminants such as cattle, sheep, deer, yak, buffalo and goats are of great importance for the production of meat and dairy products, wool, and leather. Globally, ruminants are maintained under a diverse range of farming systems and environments, and are fed a wide variety of diets. Ruminants have a complex digestive system, and digestion of feed takes place initially in the rumen. There, microbes play a key role in the breakdown of feed components such as fibre, producing short chain fatty acids that provide energy for the host. Rumen microbes are thus essential providers of animal energy and nutrition, and play a key role in the productivity and health of ruminants. Rumen archaea produce the greenhouse gas methane as a metabolic end product. This methane gas is eructated by ruminants and represents 2 to 12% dietary gross energy lost to the animal [1]. Understanding the functions and compositions of rumen microbial communities is required to improve animal productivity and to reduce the amount of energy lost as methane.

The rumen microbial community is highly complex. There are approximately 10^11^ microbial cells per gram of rumen contents and these belong to many different species and genera of bacteria, archaea [2], fungi, ciliate protozoa [3] and viruses [4]. To date, relatively few of these have been successfully cultured and characterised. Molecular analyses of rumen microbial communities allow as-yet uncultivated microbes to be detected, and have become essential tools to determine shifts that occur within microbial communities, for example, during changes in diet. The development of high-throughput sequencing techniques has made detailed microbial analyses of large-scale trials feasible, allowing subtle effects on microbial community composition to be detected as changes in absolute and relative numbers of microbial marker loci.

DNA of sufficient yield and quality is the crucial starting material for these analyses. Microbial inhabitants of the rumen are highly diverse and not all DNA extraction methods work equally well for different microbial groups. To date, several studies have shown that the DNA extraction method used has an impact on the microbial community representation in samples from different habitats [5–9], including the rumen [10]. The sampling technique (e.g. oral stomach tubing and collecting through a rumen fistula) and rumen sample fractionation (into e.g. liquid and solid) can also have an impact on microbial community parameters [3,11–13].

To enable the direct comparison of rumen community structure data from studies conducted in different laboratories and around the world, it is crucial that the rumen sampling, sample fractionation and DNA extraction methods are standardised, or at least have been shown to produce similar results. The aim of this study was to systematically compare a variety of different DNA extraction methods and their impacts on the downstream analysis of rumen microbial communities using molecular ecological methods. To do this, the quality and quantity of DNA obtained by the different methods was compared, as were bacterial, archaeal, ciliate protozoal and fungal abundances and community compositions based on microbial marker loci. In addition, the effect on microbial community composition of sampling the rumen through a fistula or using an oral stomach tube and the effect of sample fractionation was investigated. The overall goal was to find comparable and simple methods that 1) deliver high quality DNA for the majority of microbial groups, 2) are suitable for different molecular microbial ecology analyses, 3) will be suitable for use in large cohort studies, and 4) are easily transferable to other researchers.

## Materials and Methods

### Ethics statement

The use of animals, including welfare, husbandry, experimental procedures, and the collection of rumen samples used for this study, was approved by the AgResearch Grasslands (Palmerston North, New Zealand) and AgResearch Ruakura (Hamilton, New Zealand) Animal Ethics Committees, and complied with the institutional Codes of Ethical Conduct for the Use of Animals in Research, Testing and Teaching, as prescribed in the Animal Welfare Act of 1999 and its amendments (New Zealand). Rumen samples were collected as part of a series of trials conducted in New Zealand under permit numbers 12002, 12174 and 12391 (AgResearch, Grasslands Research Centre, Palmerston North), and 11897 (AgResearch, Ruakura Research Centre, Hamilton). The animals were kept at AgResearch’s Grasslands Research Centre and at Lye farm, Hamilton. Rumen samples were collected according to protocols approved in the relevant Animal Ethics approvals by sampling through a fistula or via stomach tubing.

### Comparison of DNA extraction methods

Rumen contents were collected in September of 2009 from a ruminally-fistulated Friesian-Jersey cross cow (*Bos taurus*, AgResearch animal identifier 723) and a Romney wether (*Ovis aries*, animal identifier 5472). The previously pasture-fed cow was fed 6 kg of meadow/pasture hay per day for one week before sampling and had free access to water at all times. The wether was maintained on a pasture diet which was mainly comprised of ryegrass ( *Lolium* *perenne* ). Food was withheld from the animals for two hours before sampling. Rumen samples (about 50 g) were accurately weighed, frozen at −85°C within 30 min of sampling, and freeze-dried. Each entire freeze dried sample was homogenised with a 100 W household coffee grinder (Russell Hobbs, Mordialloc, Victoria, Australia) and stored in airtight bags at −85^°^C.

In total, 15 DNA extraction protocols were compared (Table 1). The methods chosen are used to extract DNA from rumen and faecal samples in our and other laboratories. Unless otherwise stated, all methods were carried out as outlined in the instructions provided by the authors and manufacturers. Each DNA extraction method was performed in triplicate, each with 25 to 30 mg (accurately weighed) of each freeze-dried rumen sample, unless noted otherwise. DNA extracts were dispensed into 10- to 20-µl single-use aliquots, and frozen at -20 C, to avoid repeat freeze-thawing of DNA prior to downstream analyses.

**Table 1:** DNA extraction methods used in this study.

Name	Abbreviation	Comments	Reference or manufacturer
CTAB-based method	CTAB	18 units of proteinase K (New England Biolabs Inc, Ipswich, MA, USA) in a 30 µl volume and 80 µl CTAB solution were used per sample	[32]
FastDNA® Spin Kit with buffer TC	FDTB	Followed manufacturer’s instructions	Q·BIOgene, MP Biomedicals LLC, Solon, OH, USA
FastDNA® Spin Kit with buffer Y	FDYF	Followed manufacturer’s instructions	Q·BIOgene
InstaGene™ matrix	IGMA	Followed manufacturer’s instructions	Bio-Rad, Hercules, CA, USA
NucleoSpin® based method	NUSP		[33]
Phenol-chloroform with bead beating I	PCBB		[34]
Phenol-chloroform, bead beating, with filtration kit for purification I	PCFI	As for PCBB, DNA filtered with QIAquick® PCR purification kit; QIAgen, Hilden, Germany	[18]
Phenol-chloroform with no bead beating	PCNB	As for PCBB, but omitted bead beating step	Unpublished
Phenol-chloroform, bead beating, with filtration kit for purification II	PCQI	Modified PCBB, buffers used as described, DNA filtered with QIAquick® PCR purification kit	[14]
Phenol-chloroform with bead beating II	PCSA	As for PCBB, but phenol-chloroform added prior to and used in bead beating step	
PSP®Spin Stool DNA Kit, protocol 1	PSP1	Followed manufacturer’s protocol 1	Invitek GmbH, Berlin, Germany
PSP®Spin Stool DNA Kit, protocol 1	PSP2	Followed manufacturer’s protocol 2	Invitek GmbH
QIAamp® DNA Stool Mini Kit	QIAG	Followed manufacturer’s protocol for isolation of DNA from stool for pathogen detection, employing the 95°C heating option	QIAgen, Hilden, Germany
Repeated bead beating plus column	RBBC		[10]
ZR Fecal DNA MiniPrep	ZYMO	Followed manufacturer’s instructions	ZYMO Research Corporation, Orange, CA, USA

### Comparison of bead beating times

DNA was extracted in duplicate from four rumen samples collected from cow 723 and sheep 5472 [details above], and lucerne (*Medicago sativa*) chaff-fed Romney cross sheep (animal identifiers 322 and 325) using the PCQI method. The bead beating step was performed for 1, 2, 3, 4 or 5 min in a Mini-Beadbeater-96 (Biospec Products, Bartlesville, OK, USA) or for 45 s at 6.5 m s^-1^ in a FastPrep FP120 (MP Biomedicals, Santa Ana, CA, USA).

### Comparison of rumen sampling methods

Rumen samples were collected from 14 dairy cows fed fresh ryegrass dominant pasture by sampling using either oral stomach tubing or through the rumen fistula (see [14] for more details on the animals). For sampling by oral stomach tubing, a stainless steel pipe (25 mm outside diameter, wall thickness 1.2 mm) measuring 520 mm in length with a 'T' handle 350 mm from one end was used to guide the lavage tube over the back of the tongue to ensure it entered the rumen. The lavage tube (19 mm outside diameter) enabled contents to be aspirated using a 400 ml syringe from the centre of the dorsal rumen, and separate observations showed sampling to be 3 to 12 cm below the surface of the rumen contents and adjacent to the fistula. When sampling through the fistula, a handful of rumen contents was taken from the mid-point of the rumen, placed in a container and a second handful of rumen contents was squeezed to obtain liquid, which was added to the same container. Samples were frozen, freeze-dried, homogenised, DNA was extracted using the PCQI method (see Table 1 for details), and the microbial community composition determined as described below.

### Comparison of total, solid and liquid rumen sample fractions

The comparisons were made between samples collected *via* the fistulae of 16 cows using the same procedure and animals plus two additional cows from the same herd as the comparison of rumen sampling methods (above) and solid and liquid fractions. Fractionation was immediately post sampling, and each sample was divided into a liquid fraction, which passed through a 0.8 mm side of square hole sieve mesh, and a solid fraction (material retained by a 0.8 mm sieve mesh). Sample fractions were frozen, freeze-dried, homogenised, DNA was extracted using the PCQI method (see Table 1 for details), and the microbial community composition determined as described below.

### Yield, purity and integrity of DNA

DNA concentrations were measured spectrophotometrically (*A*_260 nm_, NanoDrop, Thermo Fisher Scientific, Waltham, MA, USA) and fluorometrically (Quant-iT™ dsDNA Broad Range Assay Kit, Invitrogen, Carlsbad, CA, USA). The purity of DNA was assessed spectrophotometrically from *A*_260 nm_/*A*_230 nm_ and *A*_260 nm_/*A*_280 nm_ ratios to indicate possible contamination of DNA with buffer salts and organic compounds. Integrity was determined by agarose (0.5% wt/vol) gel electrophoresis (2 h, 10 × 15 cm Mini-Sub^®^ Cell GT, Bio-Rad, Hercules, CA, USA) at 60 V using 1 Kb Plus DNA Ladder and λ DNA/Hind III fragments as molecular weight markers, post-staining with SYBR^®^ Safe DNA gel stain (Invitrogen), and illumination under UV light.

### Assessing suitability of DNA for PCR-based microbial ecology applications

To determine the suitability of extracted DNA for downstream molecular ecology techniques, archaeal, bacterial, ciliate and fungal genetic marker loci were amplified as previously described [15–17], except that 30 rather than 25 extension cycles were used for archaeal 16S rRNA gene amplification. As the various DNA extraction methods resulted in differing amounts of DNA from identical quantities of starting material, the amount of DNA in each PCR was normalised based on prior fluorometric quantification. To minimise further bias between extraction methods, 0.5 or 1 ng of DNA for marker loci from fungi and ciliate protozoa, 0.2 ng for bacteria, and 0.25 ng for archaea were used in PCR. PCR products were visualised in a 1% (wt/vol) agarose gel with SYBR^®^ Safe DNA gel stain following electrophoresis.

### Quantitative real-time PCR to enumerate microbial populations

Marker loci for bacteria, archaea, ciliate protozoa and fungi were enumerated by quantitative PCR as previously described [15–17] using a SYBR Green I fluorescence kit (LightCycler 480 SYBR Green I Master or LightCycler FastStart DNA Master SYBR Green I kits, Roche, Mannheim, Germany) on a Rotor-Gene 6000 real-time rotary analyser (Corbett Life Science, Concorde, NSW, Australia). External standards were prepared by making 10-fold serial dilutions of purified plasmid DNA containing cloned marker loci amplified from DNA of pure cultures. Three to four different 10-fold dilutions of the template DNA were each amplified in duplicate, and only values that fell within the linear range (r > 0.99) of the standard curve were used in calculations.

### Assessment of the microbial community composition

Bacterial and archaeal 16S rRNA gene regions, fungal internal transcribed spacer 1 regions (ITS1) and ciliate protozoal 18S rRNA gene regions were amplified in triplicate as described previously [14,18]. Briefly, primers (Integrated DNA Technologies Inc., Coralville, IA, USA) consisted of 454 Titanium adapter sequences A (5’-CCA TCT CAT CCC TGC GTG TCT CCG ACT CAG-3’) or B (5’-CCT ATC CCC TGT GTG CCT TGG CAG TCT CAG-3’), a two base linker sequence between the bar code and the group-specific primer, and a unique 12 base error-correcting Golay bar code attached to adapter A for sample identification followed by the specific primer sequence. Amplicons from the four microbial groups were quantified fluorometrically, normalised per sample, and pooled per microbial group. A total of 1 µg DNA of each of the four resulting pools was loaded onto an agarose gel (1% wt:vol). Bands were visualized and excised under blue light transillumination, and amplicons were gel purified with the QIAquick Gel Extraction Kit (Qiagen). Bacterial, archaeal and ciliate protozoal amplicons were sequenced using 454 GS FLX Titanium chemistry at Eurofins MWG Operon (Ebersberg, Germany) or Macrogen Inc. (Seoul, South Korea). Sequencing data were submitted via the QIIME-DB Processing Pipeline (http://www.microbio.me/qiime/) and are available in public databases (DNA extraction method comparison: EBI-SRA accession numbers ERP003658-ERP003661, DNA bead beater comparison: EBI-SRA accession numbers ERP003655-ERP003657, sampling technique and fraction comparison: MG-RAST accession numbers 4491446.3-4491449.3 (AprMT, AprRT, AprRL, AprRS samples)).

### Phylogenetic analysis of pyrosequencing reads

Pyrosequence data were processed and analyzed using the QIIME software package version 1.5 [19]. Sequences over 200 bp in length with an average quality score over 25 were assigned to a specific sample via 12 base error-correcting Golay bar codes (Table S1). Bacterial sequences were denoised and suspected chimera were removed using the OTUpipe function within QIIME. The number of bacterial, archaeal, ciliate protozoal and fungal sequencing reads available for analysis are summarised in Table S2. Sequence data were grouped into operational taxonomic units (OTUs) sharing over 97% (bacteria, archaea) or 100% (ciliate protozoa, fungi) sequence similarity. Sequences were assigned to phylogenetic groups by BLAST [20] of bacterial 16S rRNA genes against the Greengenes database (version February 2011 [21]), and of archaeal 16S rRNA genes, ciliate protozoal 18S rRNA genes, and fungal ITS1 genes against in-house databases [15,16,22]. Bacterial data were summarized at phylum, class, order, family and genus, ciliate protozoal data at genus, and fungal data at sub-genus levels. Archaea were summarized using a mixed taxonomic rank scheme [22].

### Data analysis

Statistical analyses were performed in GenStat for Windows (13th edition, VSN International, Hemel Hempstead, UK, www.genstat.co.uk). ANOVA in combination with Scheffe post-hoc tests was used to find significant differences between measured parameters following different DNA extraction methods. A probability of *p* < 0.05 was considered to indicate a significant difference. Quantitative PCR data were log-transformed prior to analysis. Phylogenetic groups with an average abundance of < 1% of the total community were excluded from analyses of microbial community composition data. Also, caution must be exercised when statistically comparing relative microbial community composition data, as values are dependent on one another (i.e. if one microbial group increases proportionally, others must decrease), which may lead to an overestimation of the significance of differences.

Spearman’s Rank and Pearson correlations between the different DNA extraction methods were calculated for bacterial (phylum, family and genus level), archaeal (mixed taxonomic ranks), fungal (sub-genus level) and ciliate protozoal (genus level) community compositions to compare extraction methods (the average of triplicate community composition determinations were used for this analysis). In addition, for each rumen sample and microbial group, a matrix of pair-wise Pearson similarities was constructed, tabulating the similarity of the community structure determined using each different DNA extraction method with the structure determined using every other method. These matrices were converted to distances, where distance = 1 – similarity, to produce six matrices (bacterial genera [sheep and cow], archaeal mixed taxonomic ranks [sheep and cow], ciliate protozoal genera [cow only], and fungal subgenera [cow only]), which were used to generate six trees using the UPGMA algorithm [23], from which a consensus tree was formed using the CONSENSE program in PHYLIP [23].

Principal coordinate analysis of Bray-Curtis dissimilarity matrices of microbial community composition data was performed in QIIME.

Differences between rumen sampling techniques and between total, liquid and solid rumen sample fractions were assessed using dependent sample *t*-tests.

## Results and Discussion

The aim of this study was to determine the effects that 15 different DNA extraction, two rumen sampling methods, and sample fractionation had on parameters such as DNA quality and quantity, as well as on absolute microbial numbers and relative microbial community composition.

### DNA yield and quality using 15 DNA extraction methods

We selected 15 different DNA extractions methods that were commercially available, published in the literature, or being used in our laboratory (Table 1). DNA was extracted, in triplicate for all 15 methods, from a single sample of freeze-dried rumen contents from a hay-fed cow and from one batch from a pasture-grazed sheep, for a total of 90 extractions. The quantity, purity, integrity and size of the DNA and its amenability to PCR amplification were assessed (Table 2). As found by others [7,24], the specific yield (g DNA/g sample) of DNA varied, depending on the DNA extraction method used. FDYF, FDTB and IGMA kits did not yield DNA that could be quantified by spectrophotometry. The PCSA and RBBC methods produced the largest specific yields of DNA, while the smallest specific yields were extracted using the NUSP, FDTB, and FDYF methods. The apparent DNA yield determined using spectrophotometry was correlated with the yield determined using fluorometry (Pearson correlation coefficient *r* = 0.915 and 0.806 for cow and sheep samples, respectively, *p* < 0.001, using each triplicate extraction as a discrete pair of data points). However, the DNA yields determined by fluorometry were higher than those determined using spectrophotometry. This may be because particulate material interfered with spectrophotometric readings, or may be because fluorescence is quenched by reagents or co-extracted rumen material in the DNA preparations. In an effort to increase the absolute yields, 100 mg of freeze dried samples were also used, but these appeared to “overload” some DNA extraction methods, and proportionally less DNA was extracted than for the standard 25 to 30 mg samples (data not shown). A similar observation was made by Ariefdjohan and colleagues [9], who found that specific yields of DNA increased when less faecal material was used for DNA extraction. Due to the particulate nature of homogenised, freeze-dried rumen samples, we decided it was not desirable to use samples of < 25 mg to avoid introducing variability due to within-sample heterogeneity.

**Table 2:** Quantities and qualities of DNA extracted with different DNA extraction methods.

Sample	Method^a^	Apparent specific DNA yield (μg g^-1^ dry weight rumen contents)^b^		DNA quality			
		Fluoro-metry	Spectro-photo-metry	*A*_260/280 nm_	*A*_260/230 nm_	Integrity^c^	PCR^d^
cow	CTAB	262	1285	1.62	1.06	+++^e^	−
	FDTB	55	n. d. ^f^	n. d.	n. d.	−	+
	FDYF	5	n. d.	n. d.	n. d.	−	+
	IGMA	184	n. d.	n. d.	n. d.	+	−
	NUSP	0	2	3.01	0.88	−	+
	PCBB^g^	281	1376	1.92	1.96	++	+++
	PCFI^g^	79	105	1.86	2.06	++	+++
	PCNB	175	443	1.54	0.88	+	+
	PCQI^g^	687	730	1.86	2.42	++	+++
	PCSA^g^	3216	5697	1.95	2.08	++	+++
	PSP1^g^	107	465	1.88	1.41	+++	+++
	PSP2^g^	134	874	1.95	1.56	+++	+++
	QIAG^g^	61	234	1.64	0.90	+++	+++
	RBBC^g^	463	2156	1.69	1.39	+++	+++
	ZYMO^g^	240	651	1.56	0.50	++	+++
sheep	CTAB	49	1369	1.66	1.21	++	−
	FDTB	139	n. d.	n. d.	n. d.	+	+
	FDYF	95	n. d.	n. d.	n. d.	+	+
	IGMA	177	n. d.	n. d.	n. d.	+	−
	NUSP	0	5	1.81	0.81	++	+
	PCBB^g^	1053	5497	1.96	1.92	+++	+++
	PCFI^g^	809	824	1.94	2.40	+++	+++
	PCNB	272	1299	1.69	0.98	++	+
	PCQI^g^	808	824	1.87	2.46	+++	+++
	PCSA^g^	7653	21323	1.87	2.05	+++	+++
	PSP1^g^	226	1073	1.98	1.87	+++	+++
	PSP2^g^	209	1768	1.98	1.87	+++	+++
	QIAG^g^	240	651	2.02	1.72	+++	+
	RBBC^g^	1315	4724	1.94	2.24	+++	+++
	ZYMO^g^	310	528	1.61	0.57	++	+++

^a^ See Table 1 for details of DNA extraction methods. ^b^ DNA extractions were performed in triplicate on each sample using each method. The values given are means of these determinations. ^c^ DNA integrity was assessed by gel electrophoresis. ^d^ Suitability for PCR was based on successful amplification of bacterial, ciliate protozoal, fungal and archaeal marker loci. ^e^ Quality criteria are scored as good (+++) , acceptable (++) , poor (+) , and unacceptable (−) ^f^ n. d. no reliable data, ^g^ Used for further investigations.

Rumen content samples contain many substances, such as tannins [25], that could inhibit the PCR [26]. Relative absorbance readings (*A*_260/230 nm_ for carbohydrates, aromatic compounds, humic acids, phenolics; *A*_260/280 nm_ for protein) provide an indication of DNA purity and should ideally be 2.0 to 2.2 for *A*_260/230 nm_ and 1.8 for *A*_260/280 nm_ for most molecular biology applications. Of all the methods tested, only the phenol chloroform-based methods PCBB and PCSA fulfilled these criteria for both the cow and sheep samples, meaning that DNA obtained using other methods may require additional purification, depending on the purpose for which it will be used.

The PSP1, PSP2, and QIAG methods extracted the highest molecular weight DNA of all methods tests, whereas those that included a mechanical lysis step such as PCSA and PCBB tended to result in extracts with more sheared DNA (data not shown). DNA extracted from the hay-fed cow rumen sample was generally more intact than that extracted from the pasture-fed sheep sample. Rumen contents from the cow fed hay comprised intact particles that appeared fibrous, whereas those from the sheep fed fresh pasture appeared less fibrous, as the particles had been damaged by chewing and were surrounded by a viscous liquid phase. DNA extracted using all methods was generally over 1 kb in size, and so should be useful for methods that generate smaller sequences, such as marker gene amplification for community structure comparisons.

With some exceptions, PCR products were amplified from all DNA extracts using primer pairs targeting marker loci in bacteria, archaea, ciliate protozoa and rumen fungi. Marker genes from fungal and ciliate protozoa could not be reliably amplified from the sheep sample using different primer combinations [15,16,18]. This was found for all extraction methods, and so was presumably due to a low prevalence of these microorganisms in this sample. We could not amplify marker genes for any of the four microbial groups from DNA extracts obtained using the CTAB method. Several modifications were made to the PCR conditions in attempts to obtain PCR products from all of these DNA preparations from the CTAB method, but all were unsuccessful. These modifications included using higher DNA concentrations, diluting template DNA to reduce the effect of potential PCR inhibitors, and increasing the number of PCR cycles to 40.

Based on the quantity and quality of extracted DNA, some methods were deemed unsuitable for extracting DNA from rumen samples and were eliminated from the remainder of our investigation. Nine methods that did result in DNA of sufficient quality and quantity for our purposes were compared using quantitative PCR and apparent microbial community structure determination by pyrosequencing. These nine were PCBB, PCFI, PCQI, PCSA, PSP1, PSP2, QIAG, RBBC, and ZYMO.

### qPCR of bacteria, archaea, fungi and ciliate protozoa

**Figure 1:**
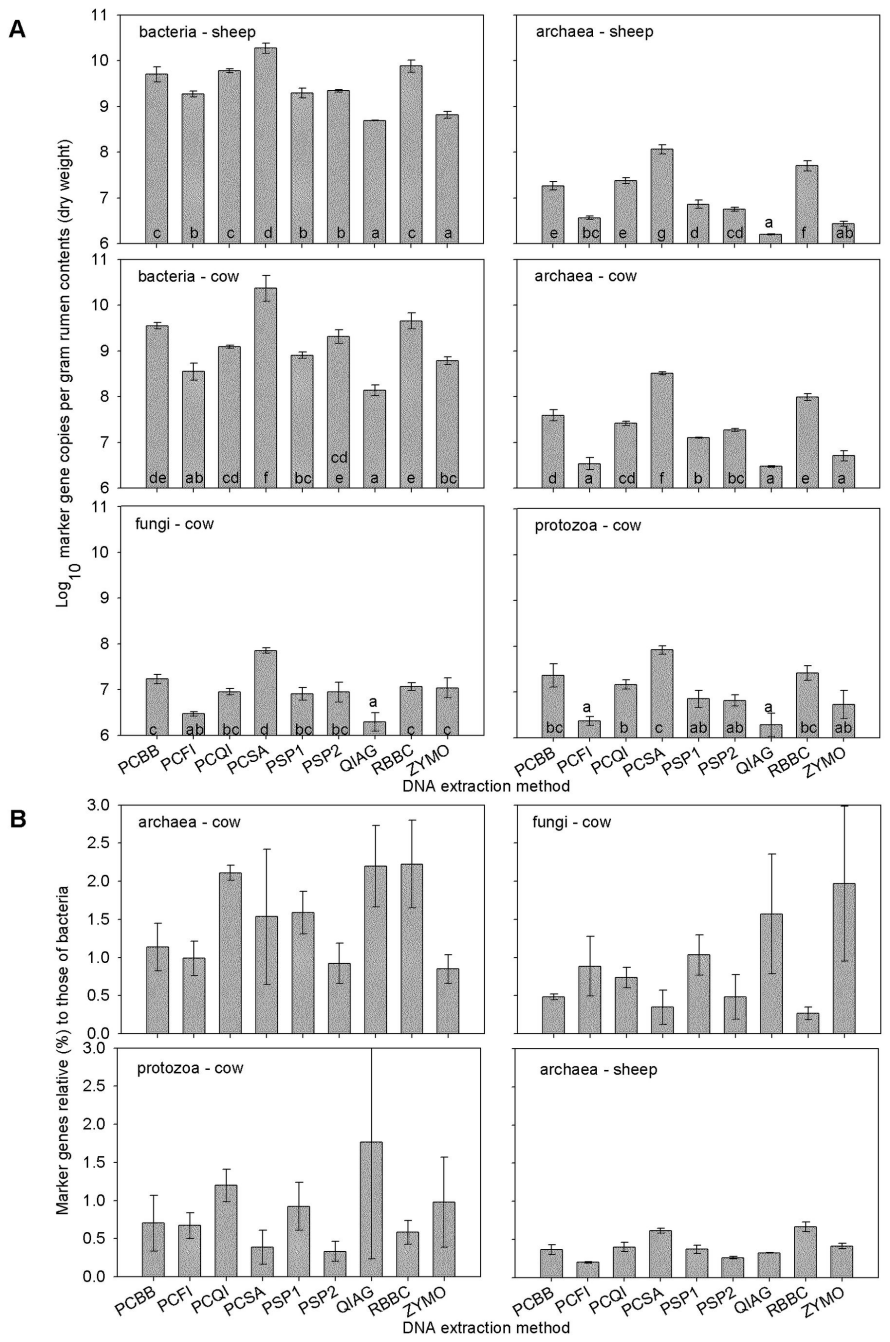
Absolute (A) and relative (B) bacterial, archaeal, fungal and ciliate protozoal marker loci copy numbers. Absolute numbers are expressed per gram dry weight of rumen contents collected from a hay-fed cow and a pasture-fed sheep from which DNA was extracted in triplicate using nine different methods (Table 1). Relative numbers are shown as a proportion of bacterial locus copies. Values depicted are means and standard deviations of log-transformed data. The vertical bars indicate one standard deviation. Those that do not share a letter at the base of the bar are significantly different (*p* < 0.05, ANOVA, Scheffe post hoc test).

To assess the quantitative effects of the nine selected DNA extraction methods on apparent microbial community structure, the numbers of bacterial, archaeal, ciliate protozoal and fungal marker genes in different DNA extracts were compared using qPCR (Figure 1). We found that the choice of DNA extraction method had a significant impact on the number of microbes detected by qPCR. Individual methods varied in terms of technical reproducibility, but the variation between triplicate replications was less than the variation between DNA extracted with different methods. The highest absolute bacterial, archaeal, fungal and ciliate protozoal gene copy numbers were consistently obtained using the PCSA and RBBC methods in both the sheep and cow samples. Absolute gene copy numbers differed over 100-fold, depending on the DNA extraction method used. Such differences have been observed in other studies of microbial communities [7,24]. We found that the number of gene copies per gram of original sample obtained by qPCR correlated with the total yield of DNA extracted (cow: *r* = 0.866, 0.962, 0.935 and 0.901 for bacteria, archaea, fungi and ciliate protozoa, respectively; sheep: *r* = 0.937 for bacteria and 0.945 for archaea, *p* < 0.001), showing that DNA quantity rather than quality was the cause of this variation. We found that archaeal, fungal and ciliate protozoal marker gene copy numbers expressed relative to bacteria differed over three-fold between different DNA extraction methods. Similar observations for *Methanomicrobiales* were noted by Bergman and colleagues [7] who found that this order represented from 9 to 95% of the total archaeal community, depending on the DNA extraction method used. This means the efficiency of the DNA extraction may differ between bacterial, archaeal, fungal and protozoal communities, and thus may result in these populations being over- or under-represented, depending on the DNA extraction method used.

### Community compositions using different DNA preparations

**Figure 2:**
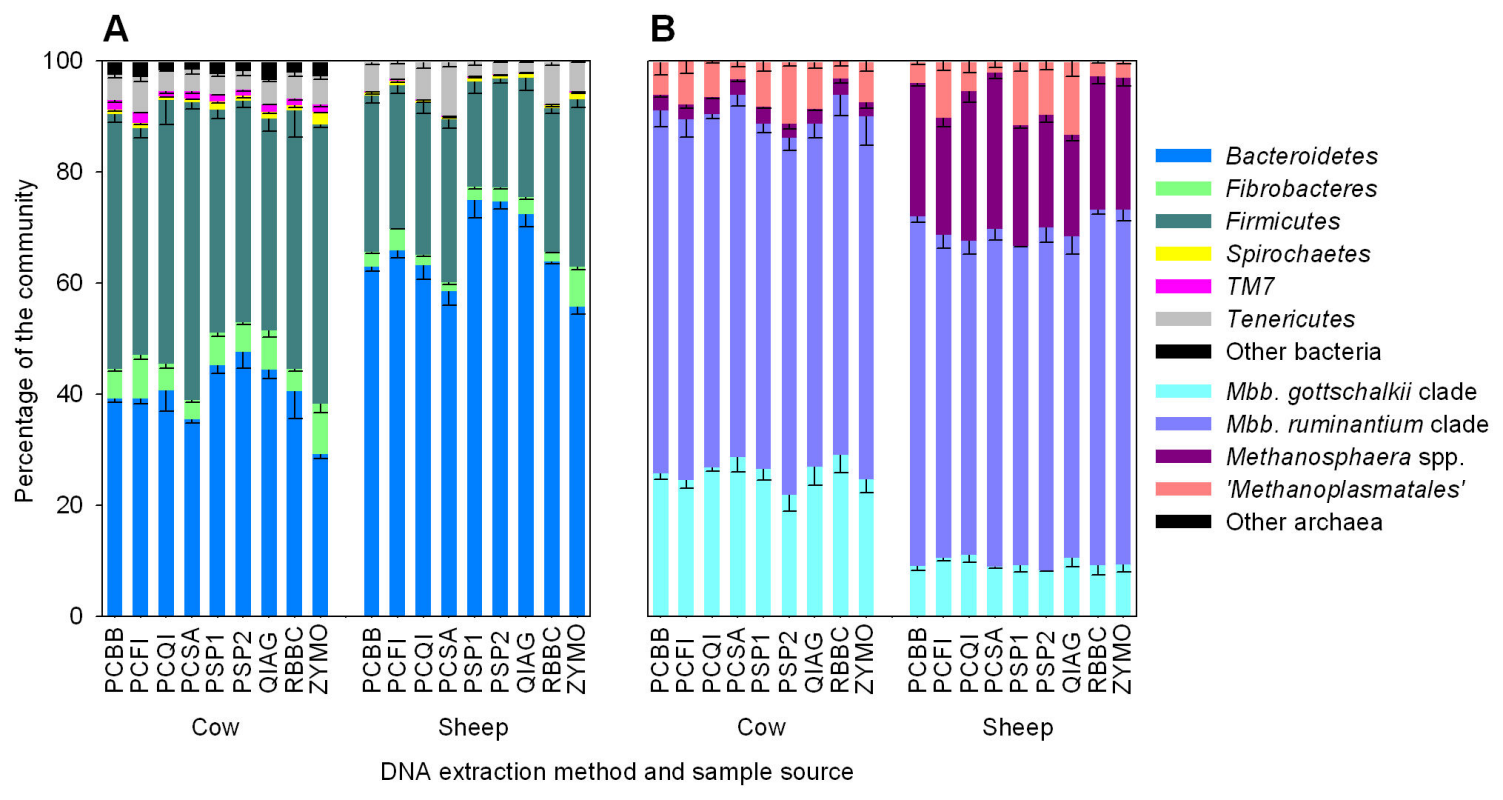
Relative (A) bacterial and (B) archaeal community compositions in rumen samples. The means (% of total community) and standard deviations, from the triplicate determinations, of the relative contribution of each microbial group in DNA obtained using the nine best extraction method are shown. The keys to the right indicate the major community components. The underlying data for bacteria, archaea, fungi and ciliate protozoa are provided in Table S3. *Mbb*, *Methanobrevibacter* .

Pyrosequencing of marker loci was used to compare apparent bacterial, archaeal, ciliate protozoal and fungal microbial community compositions in DNA obtained with the nine selected DNA extraction methods. It is important to stress that the actual community composition in the rumen samples is not known. Comparisons of DNA extracts from model communities have been made [27], but doing that in our study would not replicate the complex community and its intimate interaction with the feed matrix found in the rumen. The relative abundances of microbial groups were compared and contrasted for each DNA extraction method (Figure 2, Table S3). Importantly, all dominant microbial groups with a relative abundance over 1% were detected using all DNA extraction methods. However, the DNA extraction method did influence the apparent contribution of these groups to the community.

#### Bacteria

The choice of DNA extraction method significantly affected the abundance of bacterial groups at various taxonomic ranks. At the phylum level, *Bacteroidetes* were relatively less abundant by ZYMO and PCSA, as compared to the other methods, whereas QIAG, PSP2 and PSP1 methods resulted in relatively more *Bacteroidetes* in both the cow and sheep samples (cow: 29.3 to 47.6%, sheep: 55.8 to 75%). In contrast, the phylum *Firmicutes* was significantly more abundant when bead beating-based DNA extraction methods PCBB, PCQI, PCSA and RBBC and the ZYMO method were employed (cow: 38.1 to 53.6% relative abundance, sheep: 19 to 29.4% relative abundance). *Fibrobacteres* varied up to 2.6-fold in cows (3.5 to 9.1%) and 4.8-fold in sheep (1.5 to 7.2%) with non-bead beating methods yielding significantly greater representation of DNA from *Fibrobacteres*. Use of the ZYMO method resulted in the highest relative abundance of the subdominant phylum *Spirochaetes* (2.3 and 1.2% in the cow and sheep, respectively). Similar effects were also seen at the class, order, family and genus levels (Table S3).

#### Archaea

The choice of DNA extraction method significantly affected the representation of some archaeal groups, especially the ‘*Methanoplasmatales*’ ( [28]; also known as Rumen Cluster C [22] group). Up to a 6.6-fold difference in the relative abundance of ‘*Methanoplasmatales*’ was observed (2 to 13.2%). In general, bead beating methods such as RBBC and PCSA tended to yield fewer ‘*Methanoplasmatales*’, whereas less physical methods such as PSP2 tended to result in more ‘*Methanoplasmatales*’. This may be because these organisms are more readily lysed, but this remains to be demonstrated. The opposite was observed for sequences that fell into the *Methanosphaera* group, which apparently require greater disruption to release their DNA. The abundance of sequences belonging to the *Methanobrevibacter ruminantium* clade was similar for all methods. Sequences affiliated with the *Methanobrevibacter* *gottschalkii* clade varied up to 1.3-fold between methods.

#### Ciliate protozoa

The choice of DNA extraction method subtly affected the representation of some ciliate protozoal groups in the cow sample. The abundance of *Entodinium* varied up to 1.4-fold (32.2 to 45.7%) and *Entodinium* were relatively more abundant in DNA extracted using the PCFI, PCQI, PCSA and QIAG methods, whereas their representation was lowest when the PCBB and ZYMO methods were employed. The abundance of *Eremoplastron*-*Diploplastron* differed considerably between the ZYMO, PCFI and QIAG methods. The relative proportions of *Polyplastron* were similar for all DNA extraction methods.

#### Fungi

Overall, fungal community composition was not strongly affected by the DNA extraction method. Although relatively few sequence reads (124 to 168 reads per sample) were obtained, some subtle differences in the abundances of *Orpinomyces*, *Piromyces* and the group SK3 were observed between DNA extraction methods.

#### Overall correlations and differences

An increase in the abundance of the phylum *Firmicutes* correlated with a decrease in the abundance of *Bacteroidetes* (cow: *r* = -0.805; sheep: *r* = -0.976, *p* < 0.001). Such DNA extraction-dependent correlations between *Firmicutes* and *Bacteroidetes* have previously been observed [6]. Interestingly, the total amount of DNA extracted tended to be positively correlated with the abundance of the phylum *Firmicutes* (cow: *r* = 0.669, *p* < 0.001; sheep: *r* = 0.435, *p* = 0.023) and negatively correlated with the abundance of the phylum *Bacteroidetes* (cow: *r* = -0.325, *p* = 0.098; sheep: *r* = -0.455, *p* = 0.017). Although it not possible to determine which method extracted DNA most representative of the rumen microbial community, these data do indicate that methods without a mechanical lysis step extract less DNA. This may generate bias towards the Gram-negative *Bacteroidetes*, which are probably more readily lysed than the Gram-positive *Firmicutes* [29]. It is noteworthy that, for the sheep sample, significantly fewer pyrosequencing reads were retained following quality filtering and chimera removal by OTUpipe for those methods that involved bead beating (PCSA, PCBB and RBBC; Table S2), especially when compared to kit-based methods such as QIAG that do not harshly mechanically lyse the cells. The sample taken from the grass-fed sheep was less fibrous in nature than the sample from the hay-fed cow. This may indicate that the DNA extracted from the sheep sample was more fragmented following bead beating, which would result in more spurious amplifications in downstream PCR steps, such as the formation of chimeric amplicons. It is therefore important not to subject samples to overly harsh mechanical lysis, as there is a trade-off between obtaining sufficient DNA that is representative of the rumen microbial community and damaging the DNA in the process of extracting it.

### Comparison of DNA extraction methods

**Figure 3:**
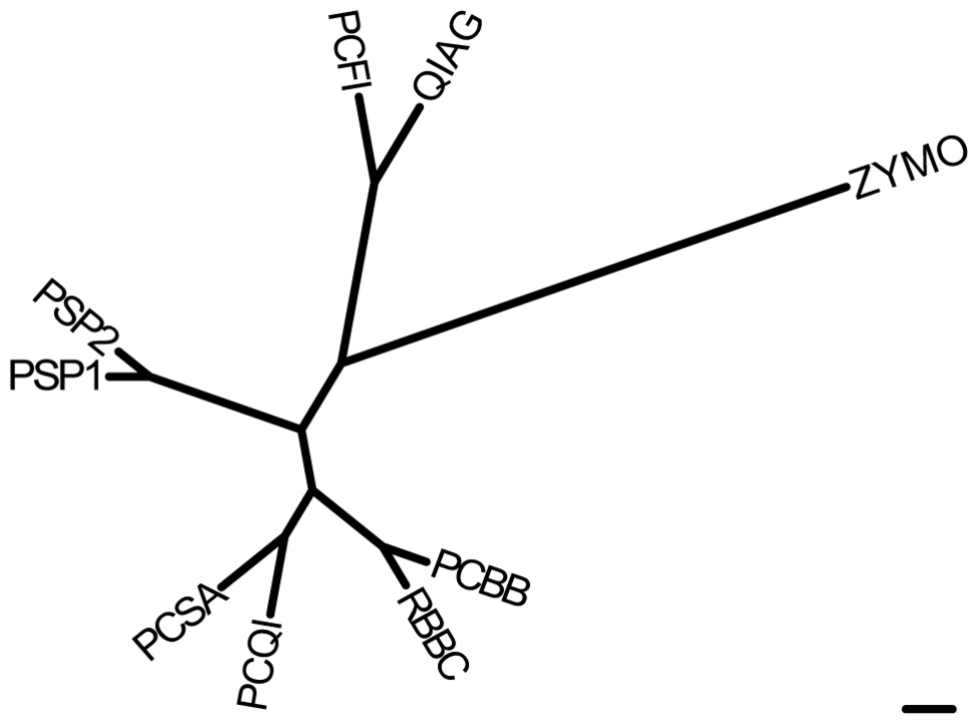
Consensus dendrogram illustrating the similarity of microbial communities obtained using different DNA extraction methods. For each rumen sample and microbial group, a matrix of pair-wise Pearson similarities was constructed, tabulating the similarity of the community structure determined using each different DNA extraction method with the structure determined using DNA from every other method. These matrices were converted to distances to produce six matrices (bacterial genera [sheep and cow], archaeal mixed taxonomic ranks [sheep and cow], ciliate protozoal genera [cow only], and fungal subgenera [cow only]), which were used to generate six trees using the UPGMA algorithm [23], from which a consensus tree was formed using the CONSENSE program in PHYLIP [23]. The scale bar represents 1% community difference.

Spearman’s Rank and Pearson correlations between the different DNA extraction methods were calculated for bacterial, archaeal, ciliate protozoal and fungal communities (Table S4). On the whole, good correlations between apparent microbial composition data from different DNA extraction methods were observed. The Pearson correlation coefficient *r* ranged from 1.000 for several methods to 0.823, which was the lowest Pearson correlation observed and was between the ZYMO and the PSP2 methods when cow bacterial compositions were compared at family level. The PCQI and RBBC methods correlated well, the lowest correlation being 0.978 for ciliate protozoa in the cow and correlations of 0.9995 and 0.9996 being observed for bacteria at phylum level in both the sheep and the cow, respectively. To determine which specific microbial taxa were potentially most affected by a given DNA extraction method, ANOVA in combination with the conservative Scheffe *post hoc* test was performed (Table S5). From these analyses, it was concluded that apparent microbial community structures determined using the PCQI and RBBC methods were generally comparable, whereas for others, such as PSP1 and ZYMO, the relative abundances differed significantly for many microbial taxa. This is also displayed graphically in Figure 3, which illustrates the apparent similarity of microbial communities obtained using different DNA extraction methods.

**Figure 4:**
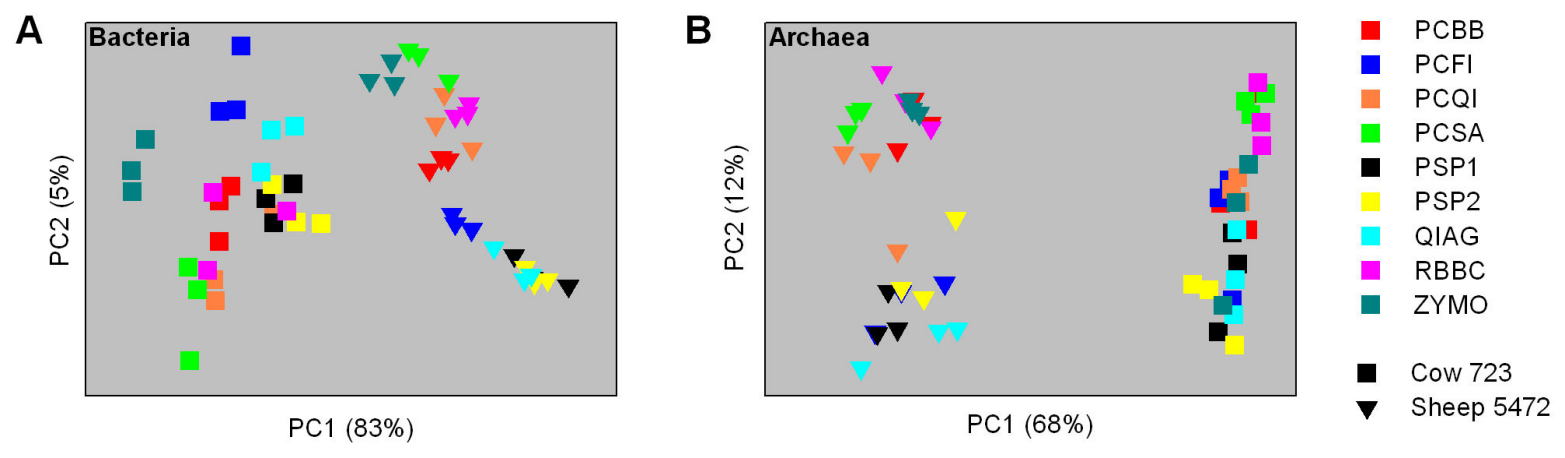
Comparison of microbial community compositions following extraction of DNA with different methods. Principal coordinate analyses of Bray-Curtis dissimilarities of A) bacterial communities (phylum level) and B) archaeal communities (mixed taxonomic ranks) in cow and sheep rumen samples are shown. The data from each of the individual triplicate extractions performed are plotted. The keys to the right indicate the different DNA extraction methods used. The values in parentheses give the amount of variation explained by each coordinate.

Principal coordinate analysis was used to compare and contrast the apparent compositions of microbial communities obtained from different DNA extraction methods (Figure 4). The bacterial community composition varied between DNA extraction methods. Data points from different DNA extraction methods did not always group closely, indicating that the methods do not retrieve the same components of the community with equal efficiency. There was some close grouping of data points from the RBBC and PCBB methods, as well as the PSP1, PSP2 and QIAG methods. Data points obtained from the sheep sample were much more widely spread than those of the cow sample, indicating that the choice of DNA extraction method had a greater effect on the perceived sheep bacterial community. Archaeal community composition also varied between DNA extraction methods. Again, no methods appeared to be directly comparable, although there was some overlap and/or proximity of data points for PSP1, PCFI and QIAG, as well as RBBC and PCSA methods for both the sheep and the cow samples. Similar to the archaea and bacteria, the ciliate protozoal community composition also varied between DNA extraction methods (data not shown).

### Comparison of bead beating times

Bead beating is frequently used to lyse bacterial cells. The impact of the degree of bead beating on DNA yield and the microbial community composition was assessed on rumen samples from four different animals (Table S6). The greatest yield of DNA was obtained when samples were mechanically disrupted for 4 or 5 min with the Mini-Beadbeater-96 or the FastPrep FP120. Members of the archaeal order ‘*Methanoplasmatales*’ and of the bacterial the phylum *Bacteroidetes* were less abundant when mechanical disruption was prolonged (4 or 5 min). Concomitantly, members of the bacterial phylum *Firmicutes* were more abundant, possibly indicating that these cells are harder to lyse. DNA yields increased with increasing bead-beating time, suggesting that more difficult to lyse cells were being disrupted. However, overall, the bacterial, archaeal and ciliate protozoal community compositions were similar, despite different levels of mechanical disruption. Principal coordinate analyses showed that the points for each sample grouped together, regardless of the bead beating technique used (Figure S1). In the interest of fragmenting the DNA as little as possible, whilst still obtaining a high and representative yield of DNA, we recommend using the Mini-Beadbeater-96 for 4 min to mechanically lyse microbes in rumen samples.

### Microbial communities in samples collected with different methods

**Figure 5:**
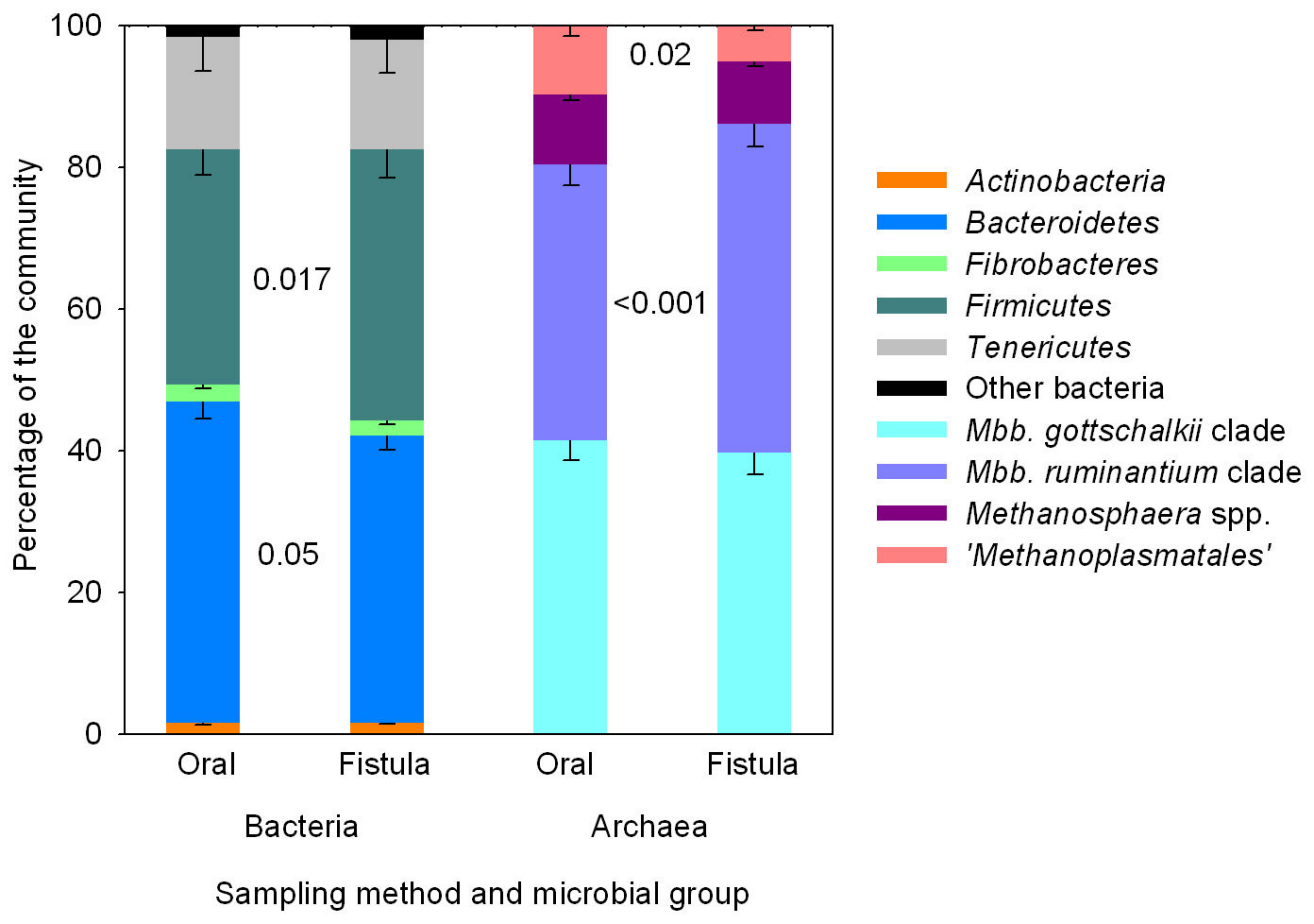
Impact of rumen sampling method on the (A) bacterial and (B) archaeal rumen microbiota composition. The apparent microbial community structure in parallel samples collected from 14 cattle using an oral stomach tube or through a rumen fistula was compared. The means (% of total community) and standard errors of the relative contribution of each microbial group are shown. The keys to the right indicate the major community components. The underlying data for bacteria, archaea, fungi and ciliate protozoa are provided in Table S7. *Mbb*, *Methanobrevibacter* . Differences between rumen sampling methods were assessed using dependent sample *t*-tests.

We compared apparent microbial community structure in parallel samples collected from 14 cattle using an oral stomach tube or through a rumen fistula. These are two commonly used methods for sampling rumen contents. The apparent bacterial, archaeal, fungal and ciliate protozoal microbial community composition in these rumen samples was compared (Figure 5, Table S7). Differences in the relative abundances of some microbial groups were observed between the two sampling methods. Sampling *via* oral stomach tube appeared to enhance the abundance of the family *Prevotellaceae* (1.3-fold increase, 24.8 to 31.3%) but decrease the abundance of the family *Lachnospiraceae* (1.4-fold decrease, 14.5 to 20.1%), including the affiliated genera *Butyrivibrio* and *Coprococcus* . Within the domain *Archaea*, the abundance of sequences affiliated with the *Methanobrevibacter ruminantium* clade were 1.2-fold (38.9 to 46.3%) more abundant in samples collected *via* the rumen fistula, whereas sequences belonging to the ‘*Methanoplasmatales*’ were 1.9-fold more prevalent (5.1 to 9.6%) in samples collected via an oral stomach tube. Similar effects were seen with several members of the ciliate protozoal community. For example, the dominant genus, *Epidinium*, was relatively more abundant when samples were taken *via* the rumen fistula, while sequences belonging to the genera *Entodinium*, *Eudiplodinium* and the *Eremoplastron-Diploplastron* group were relatively more abundant when samples were obtained by oral stomach tubing. The sampling method did not seem to have a great effect on the anaerobic fungal community in these samples. Only relatively minor differences were detected, mainly within the *Caecomyces* 1, *Neocallimastix* 1, *Piromyces* 2, *Piromyces* 3, and SK3 groups, and the prevalence of some potentially novel sequences that were not closely related to reference sequences in the GenBank database. Despite differences in abundance detected for specific taxa, samples collected *via* the fistula or *via* an oral stomach tube could not be readily distinguished by principal coordinate analysis (data not shown). In a previous study, the bacterial diversity in rumen fluid samples obtained *via* a rumen fistula and *via* oral stomach tubing from three sheep fed chopped lucerne and two cows fed hay was compared using denaturing gradient gel electrophoresis analysis of bacterial 16S rRNA genes [30]. As in our study, the bacterial communities clustered by animal and ruminant species rather than by sampling method, suggesting that differences between sampling methods were negligible for this comparison.

Despite the overall resemblance of microbial community structure derived from the samples collected with the two different methods, it should not be overlooked that the relative abundance of certain microbial groups appeared to differ significantly depending on the method used. The differences in the relative abundance of certain taxa may be explained by the size of the tube used. Oral stomach tubing only allows for small, highly degraded pieces of fibre to be sampled. Thus, primary colonizers of ingested plant material may be under-represented by this sampling technique. Secondly, the rumen consists of several micro-niches that vary in their chemical [31] and physical characteristics, and may therefore also vary in their microbial community composition. Sampling through the fistula allows consistent sampling of a similar site; however the placement of the oral stomach tube in the rumen *via* the oesophagus cannot be influenced easily [11]. Thus, results obtained from samples collected by oral stomach tubing may additionally be influenced by the specific rumen location sampled [31]. As a consequence, statistically significant differences in microbial community structure between hosts showing different phenotypes such as low and high feed conversion efficiency cattle may be more difficult to detect, but it is often possible to sample a greater number of intact, compared to fistulated animals.

Overall, community structure differences were minor when compared to differences introduced by the choice of DNA extraction method. Samples obtained through the fistula and via oral stomach tubing both give an equally valid qualitative representation of microbial community structure. Additionally, sampling taken by stomach tube allows an easier assessment of differences in rumen microbial communities in individuals that have been selected for desirable traits (e.g. efficiency of feed use for production, resistance to disease or toxins) in commercial herds and flocks. This technique enables large numbers of individuals to be screened, rapidly and at low cost, and avoids the need for surgical insertion of a fistula, a procedure that is not practicable in a commercial setting and normally restricted to experimental animals.

### Comparison of total, solid and liquid rumen sample fractions

**Figure 6:**
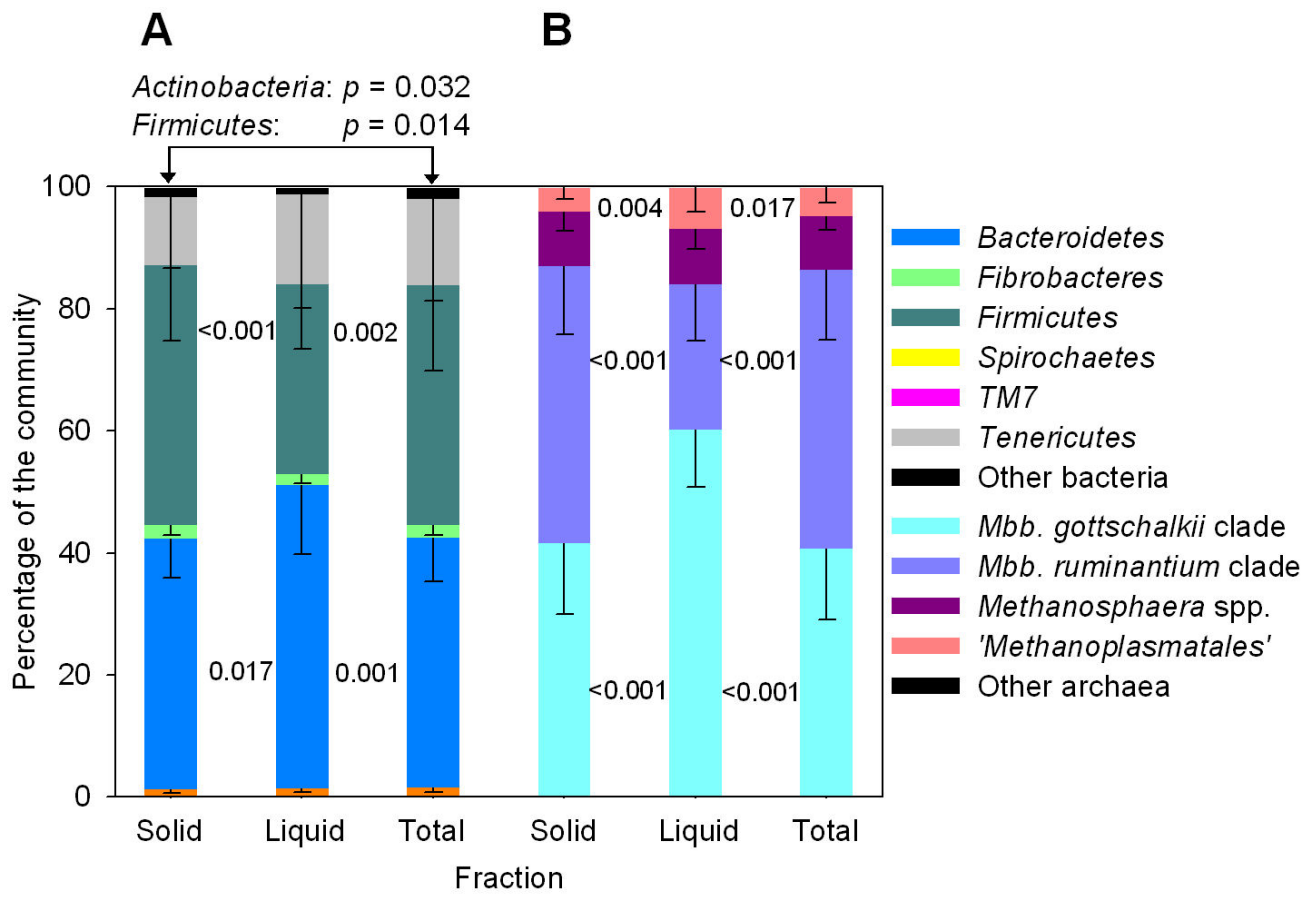
Impact of rumen sample fractionation on the (A) bacterial and (B) archaeal rumen microbiota composition. The apparent microbial community structure in samples collected from 16 cattle were separated into liquid, solid and total rumen sample fractions and the apparent microbial community structures were compared. The means (% of total community) and standard errors of the relative contribution of each microbial group are shown. The keys to the right indicate the major community components. The underlying data for bacteria, archaea, fungi and ciliate protozoa are provided in Table S8. *Mbb*, *Methanobrevibacter* . Differences between between total, liquid and solid rumen sample fractions were assessed using dependent sample *t*-tests.

Samples of rumen digesta are frequently separated into liquid and solid fractions for analysis. The apparent bacterial, archaeal, fungal and ciliate protozoal microbial community composition in total, solid and liquid rumen sample fractions from 14 to 16 dairy cattle was compared (Figure 6, Table S8). Differences in the relative abundances of some microbial groups were observed between the sample fractions. We found that the liquid fraction appeared to contain a higher relative abundance of the family *Prevotellaceae* (1.4-fold increase, 25.1 to 36.1%) and a lower abundance of the family *Lachnospiraceae* (1.6-fold decrease, 12.7 to 20.8%) when compared with total (and solid) rumen sample fraction. These findings generally agree with those of others [3,12,13]. Within the domain *Archaea*, the abundance of sequences affiliated with the *Methanobrevibacter ruminantium* clade were 1.9-fold (45.7 to 23.8%) less abundant in the liquid fraction, whereas sequences belonging to the *Methanobrevibacter* *gottschalkii* clade were 1.5-fold more prevalent (40.7 to 60.1%) in liquid sample fractions. Similar effects were seen with several members of the ciliate protozoal and fungal communities. Overall, significant differences in community structure were observed between liquid, solid and total rumen fractions, and these differences may reflect the niches microbes inhabit within the rumen. Differences in microbial communities were most striking when liquid rumen sample fractions were compared with solid and total rumen sample fractions, and it appears that the liquid rumen sample fraction is not representative of the total rumen sample.

### Summary and conclusions

The study of rumen microbial community structure, and the ability to compare datasets obtained by different research groups around the world, will help understand the relationships between different components of these complex communities, their hosts and diet. However, to enable comparison between different studies, protocols for rumen sampling, DNA extraction and PCR amplification should be standardized or have been demonstrated to produce similar results. This is particularly relevant if data are to be compared on a global scale, such as will be the case with the Global Rumen Census (www.globalrumencensus.org.nz), and RuminOmics (www.ruminomics.eu) projects. Ideally, samples would be processed using an agreed sample processing and data analysis pipeline.

We found that the choice of DNA extraction method affected the apparent microbial community structure in ways that would be considered statistically and biologically significant, even though the DNA was extracted from subsamples of the same homogenised rumen sample. This was not a sub-sampling artefact, because three parallel sub-samples treated in the same way for every method tested always resulted in the similar apparent community structures. None of the DNA extraction methods resulted in 100% comparable bacterial, archaeal or ciliate protozoal community compositions, either from a compositional or numerical perspective. In fact, only a few DNA extraction methods appeared to be similar enough to allow direct comparison of most community parameters. Despite the major impact the DNA extraction had, variation between the hay-fed cow and pasture-fed sheep rumen samples was generally greater than the variation between DNA extraction methods and or replicates within the extraction method. This indicated that the disparate communities from these samples could be distinguished regardless of the chosen DNA extraction method. This may not have been possible if differences between samples were less apparent, for example when comparing individual animals on the same diet.

When choosing a DNA extraction method, other factors must also be considered, such as the quality required for downstream analysis, overall variability of the method between different researchers, as well as the availability of equipment, reagents and kits. For example, methods that minimise shearing are best for genome sequencing, those with low coefficients of variation are preferable to detect small differences in parameters, whereas those that can be scaled up will be favoured for processing of large sample numbers. For these reasons, we cannot endorse or advise against using a certain DNA extraction method. We routinely use the PCQI DNA extraction method in our laboratory. This method produces results similar to the RBBC method but has the added advantage that it can easily be adapted to a 96-well plate format, so that samples can later be processed with a robot to minimise variation, human error and reduce the time for sample preparation.

Most importantly, researchers need to be aware that apparent microbial community structures obtained from studies that used different DNA extraction methods are not necessarily comparable. This is especially relevant when designing large cohort studies or when conducting meta-analyses of data from different studies.

## Supporting Information

Figure S1 **Microbial community compositions following DNA extraction using different bead beating methods and durations.** DNA was extracted in duplicate from rumen contents of three sheep and one cow with the PCQI DNA extraction method employing different bead beating methods and times. Principal coordinate analyses of Bray-Curtis dissimilarities of A) phylum level bacterial and B) mixed taxonomic rank level archaeal communities in these cow and sheep rumen content are depicted here. The data from each of the individual duplicate extractions performed are plotted. The values in parentheses give the amount of variation explained by each coordinate.
(TIF)

Table S1 **Parameters used to assign sequences to a specific sample via 12 base error-correcting Golay bar codes.**
(XLSX)

Table S2 **Mean numbers of sequencing reads used to determine apparent rumen microbial community compositions.** The effect of DNA extraction and rumen sampling methods on apparent microbial community compositions was investigated using A) DNA extracted in triplicate from rumen contents of a hay-fed cow and B) DNA extracted in triplicate from rumen contents of a pasture-fed sheep, extracted using nine different methods (Table 1), C) DNA extracted from rumen samples collected in parallel from 14 dairy cows *via* either oral stomach tubing or a rumen fistula, and D) DNA extracted from the total, solid or liquid fractions of rumen samples collected in parallel from the 14 dairy cow plus two additional cows from the same flock *via* a rumen fistula.
(DOCX)

Table S3 **Significant effects of DNA extraction methods on the apparent rumen microbial community structure.** DNA was extracted in triplicate from rumen contents of A) a hay-fed cow and B) a pasture-fed sheep using nine different methods assessed in this study (Table 1). The mean abundances (%) of the dominant bacterial, archaeal, fungal and ciliate protozoal microbial taxa at different taxonomic ranks were calculated. SE – Standard error of differences; *p* – Probability that the abundance of microbial groups is not significantly different using the F-test. See Table S5 for *post-hoc* comparison results.
(DOCX)

Table S4 **Correlations of apparent microbial community structures following extraction of DNA with different methods.** Pearson (A, B) and Spearman’s rank (C, D) correlations between different parts of the microbial community at different taxonomic levels were calculated using mean abundances of dominant bacterial, archaeal, fungal and ciliate protozoal taxa (from data in Table S3) measured in DNA from (A, C) cow and (B, D) sheep rumen content samples extracted using nine different extraction methods (Table 1).
(DOCX)

Table S5 **Effect of DNA extraction method on the apparent rumen microbial community structure *post-hoc* values.** DNA extraction methods (Table 1) that do not share a letter are significantly different for the particular taxon in which the letters are listed (*p* < 0.05, ANOVA, Scheffe *post-hoc* test). Test was performed on the individual replicates that underlie the data in Table S3.
(DOCX)

Table S6 **Effects of bead beating methods on DNA extractions from rumen samples.** Microbial community compositions (A, % of total community), specific DNA yields (B), and the mean number of sequencing reads per sample (C). DNA was extracted in duplicate (means of duplicate determinations are shown) with the PCQI DNA extraction method from rumen contents of three sheep and one cow, employing different bead beating methods and times.
(DOCX)

Table S7 **Effect of rumen sampling method on the apparent microbial community structure.** Microbial community compositions (% of total community) from DNA extracted from rumen samples obtained by oral stomach tubing and through a fistula from 14 dairy cows.
(DOCX)

Table S8 **Effect of rumen sample fractionation on the apparent microbial community structure.** Microbial community compositions (% of total community) from rumen sample fractions (liquid, solid and total) of 16 dairy cows.
(DOCX)
