# Supplementary figures and images for: Effect of DNA Extraction Methods and Sampling Techniques on the Apparent Structure of Cow and Sheep Rumen Microbial Communities
Source: PLoS One. 2013 Sep 11;8(9):e74787. doi: 10.1371/journal.pone.0074787 (PMC3770609; doi:10.1371/journal.pone.0074787)

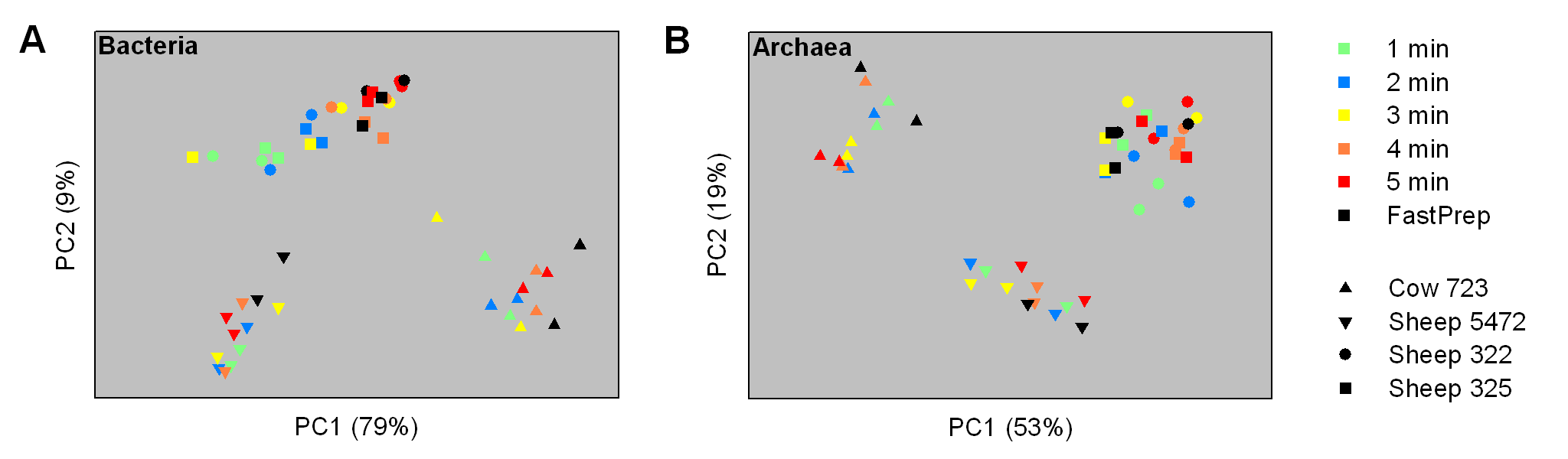

Supplement: Figure S1 — Microbial community compositions following DNA extraction using different bead beating methods and durations. DNA was extracted in duplicate from rumen contents of three sheep and one cow with the PCQI DNA extraction method employing different bead beating methods and times. Principal coordinate analyses of Bray-Curtis dissimilarities of A) phylum level bacterial and B) mixed taxonomic rank level archaeal communities in these cow and sheep rumen content are depicted here. The data from each of the individual duplicate extractions performed are plotted. The values in parentheses give the amount of variation explained by each coordinate. (TIF) [file pone.0074787.s001.tif]
