# Supplementary material for: Effect of DNA Extraction Methods and Sampling Techniques on the Apparent Structure of Cow and Sheep Rumen Microbial Communities
Source: PLoS One. 2013 Sep 11;8(9):e74787. doi: 10.1371/journal.pone.0074787 (PMC3770609; doi:10.1371/journal.pone.0074787)
Supplement: Table S2 — Mean numbers of sequencing reads used to determine apparent rumen microbial community compositions. The effect of DNA extraction and rumen sampling methods on apparent microbial community compositions was investigated using A) DNA extracted in triplicate from rumen contents of a hay-fed cow and B) DNA extracted in triplicate from rumen contents of a pasture-fed sheep, extracted using nine different methods (Table 1), C) DNA extracted from rumen samples collected in parallel from 14 dairy cows via either oral stomach tubing or a rumen fistula, and D) DNA extracted from the total, solid or liquid fractions of rumen samples collected in parallel from the 14 dairy cow plus two additional cows from the same flock via a rumen fistula. (DOCX) [file pone.0074787.s003.docx]

**Table S2. Mean numbers of sequencing reads used to determine apparent rumen microbial community compositions.**

The effect of DNA extraction and rumen sampling methods on apparent microbial community compositions was investigated using A) DNA extracted in triplicate from rumen contents of a hay-fed cow and B) DNA extracted in triplicate from rumen contents of a pasture-fed sheep, extracted using nine different methods (Table 1), C) DNA extracted from rumen samples collected in parallel from 14 dairy cows *via* either oral stomach tubing or a rumen fistula, and D) DNA extracted from the total, solid or liquid fractions of rumen samples collected in parallel from the 14 dairy cow plus two additional cows from the same flock *via* a rumen fistula.

**A. Hay-fed cow**

| Microbial group | Step | DNA extraction method | | | | | | | | | |
| --- | --- | --- | --- | --- | --- | --- | --- | --- | --- | --- | --- |
|  |  | PCBB | PCFI | PCQI | PCSA | PSP1 | PSP2 | QIAG | RBBC | ZYMO | SE^a^ |
| Bacteria | Before OTU pipe | 3130 | 3177 | 2897 | 3060 | 3631 | 3584 | 3573 | 3280 | 2777 | 384 |
|  | After OTU pipe | 2195 | 2196 | 2092 | 2088 | 2517 | 2491 | 2721 | 2457 | 2057 | 329 |
|  | % remaining | 70.3 | 69.1 | 72.1 | 68.2 | 69.2 | 69.6 | 76.3 | 73.9 | 73.9 | 3.1 |
| Archaea |  | 704 | 765 | 1718 | 718 | 718 | 689 | 693 | 763 | 831 | 135 |
| Ciliate protozoa |  | 2385 | 2467 | 2138 | 2211 | 2618 | 1971 | 1538 | 2617 | 2270 | 444 |
| Fungi |  | 139 | 140 | 132 | 143 | 168 | 147 | 124 | 164 | 159 | 11 |

^a^SE, standard error of differences of means.

**B. Pasture-fed sheep**

| Microbial group | Step | DNA extraction method | | | | | | | | | |
| --- | --- | --- | --- | --- | --- | --- | --- | --- | --- | --- | --- |
|  |  | PCBB | PCFI | PCQI | PCSA | PSP1 | PSP2 | QIAG | RBBC | ZYMO | SE^a^ |
| Bacteria | Before OTU pipe | 4127 | 3863 | 4435 | 3874 | 4020 | 3891 | 4778 | 3792 | 3991 | 300 |
|  | After OTU pipe | 3207 | 3121 | 3528 | 3001 | 3230 | 3150 | 3956 | 2981 | 3222 | 248 |
|  | % remaining | 77.7 | 80.8 | 79.6 | 77.4 | 80.3 | 80.9 | 82.7 | 78.6 | 80.8 | 0.9 |
| Archaea |  | 749 | 771 | 783 | 819 | 800 | 857 | 702 | 819 | 759 | 94.8 |

^a^SE, standard error of differences of means.

**C. Fourteen dairy cows**

| Microbial group | Rumen sampling method | | | |
| --- | --- | --- | --- | --- |
|  | Oral stomach tubing | | Fistula | |
|  | Mean reads | SE^a^ | Mean reads | SE^a^ |
| Bacteria | 8399 | 1108 | 5861 | 924 |
| Archaea | 2296 | 181 | 2492 | 139 |
| Ciliate protozoa | 1138 | 195 | 938 | 99 |
| Fungi | 2649 | 239 | 2777 | 513 |

^a^SE, standard error of differences of means.

**D. Sixteen dairy cows**

| Microbial group | Rumen sample fraction | | | | | |
| --- | --- | --- | --- | --- | --- | --- |
|  | Liquid | | Solid | | Total | |
|  | Mean reads | SE^a^ | Mean reads | SE^a^ | Mean reads | SE^a^ |
| Bacteria | 6023 | 504 | 7363 | 920 | 5726 | 889 |
| Archaea^b^ | 2271 | 127 | 2307 | 130 | 2462 | 138 |
| Ciliate protozoa | 1198 | 236 | 2051 | 433 | 944 | 92 |
| Fungi | 3130 | 253 | 1955 | 162 | 2662 | 492 |

^a^SE, standard error of differences of means, ^b^ for archaea, the comparison was made with 15 animals.
