# Supplementary material for: Effect of DNA Extraction Methods and Sampling Techniques on the Apparent Structure of Cow and Sheep Rumen Microbial Communities
Source: PLoS One. 2013 Sep 11;8(9):e74787. doi: 10.1371/journal.pone.0074787 (PMC3770609; doi:10.1371/journal.pone.0074787)
Supplement: Table S3 — Significant effects of DNA extraction methods on the apparent rumen microbial community structure. DNA was extracted in triplicate from rumen contents of A) a hay-fed cow and B) a pasture-fed sheep using nine different methods assessed in this study (Table 1). The mean abundances (%) of the dominant bacterial, archaeal, fungal and ciliate protozoal microbial taxa at different taxonomic ranks were calculated. SE – Standard error of differences; p – Probability that the abundance of microbial groups is not significantly different using the F-test. See Table S5 for post-hoc comparison results. (DOCX) [file pone.0074787.s004.docx]

**Table S3. Significant effects of DNA extraction methods on the apparent rumen microbial community structure.**

DNA was extracted in triplicate from rumen contents of A) a hay-fed cow and B) a pasture-fed sheep using nine different methods assessed in this study (Table 1). The mean abundances (%) of the dominant bacterial, archaeal, fungal and ciliate protozoal microbial taxa at different taxonomic ranks were calculated. SE – Standard error of differences; *p* – Probability that the abundance of microbial groups is not significantly different using the F-test. See Table S5 for *post-hoc* comparison results.

**A. Hay-fed cow**

| Microbial group | Taxonomic rank | Taxon | DNA extraction method | | | | | | | | | | | |
| --- | --- | --- | --- | --- | --- | --- | --- | --- | --- | --- | --- | --- | --- | --- |
|  |  |  | PCBB | PCFI | PCQI | PCSA | PSP1 | PSP2 | QIAG | RBBC | ZYMO | SE | LSD | *p* |
| Bacteria | Phylum | *Bacteroidetes* | 39.2 | 39.3 | 40.7 | 35.5 | 45.3 | 47.6 | 44.4 | 40.6 | 29.3 | 2.0 | 4.2 | <0.001 |
|  |  | *Fibrobacteres* | 5.3 | 7.8 | 4.8 | 3.5 | 5.8 | 5.4 | 7.1 | 4.1 | 9.1 | 0.7 | 1.5 | <0.001 |
|  |  | *Firmicutes* | 45.9 | 40.8 | 47.4 | 53.6 | 40.2 | 39.8 | 38.1 | 46.5 | 50.2 | 2.1 | 4.4 | <0.001 |
|  |  | *Spirochaetes* | 0.7 | 1.0 | 0.7 | 0.7 | 1.4 | 0.9 | 1.1 | 0.9 | 2.3 | 0.2 | 0.4 | <0.001 |
|  |  | TM7 | 1.7 | 1.9 | 1.0 | 1.2 | 1.4 | 1.1 | 1.4 | 1.2 | 1.4 | 0.2 | 0.5 | 0.015 |
|  |  | *Tenericutes* | 4.6 | 6.3 | 3.7 | 3.9 | 3.5 | 3.4 | 4.3 | 4.6 | 5.1 | 0.5 | 1.0 | <0.001 |
|  | Class | *Bacteroidia*^a^ | 39.2 | 39.3 | 40.7 | 35.5 | 45.3 | 47.6 | 44.4 | 40.6 | 29.3 | 2.0 | 4.2 | <0.001 |
|  |  | *Fibrobacteres*^a^ | 5.3 | 7.8 | 4.8 | 3.5 | 5.8 | 5.4 | 7.1 | 4.1 | 9.1 | 0.7 | 1.5 | <0.001 |
|  |  | *Clostridia*^a^ | 45.9 | 40.8 | 47.4 | 53.6 | 40.2 | 39.8 | 38.1 | 46.5 | 50.1 | 2.1 | 4.4 | <0.001 |
|  |  | *Spirochaetes* | 0.7 | 0.9 | 0.6 | 0.7 | 1.3 | 0.9 | 1.1 | 0.8 | 2.2 | 0.2 | 0.4 | <0.001 |
|  |  | TM7-3^a^ | 1.7 | 1.9 | 1.0 | 1.2 | 1.4 | 1.1 | 1.4 | 1.2 | 1.4 | 0.2 | 0.5 | 0.015 |
|  |  | *Erysipelotrichi* | 1.7 | 1.9 | 1.6 | 1.8 | 1.5 | 1.6 | 1.8 | 2.5 | 1.9 | 0.3 | 0.7 | 0.181 |
|  |  | *Mollicutes* | 2.9 | 4.3 | 2.1 | 2.1 | 1.9 | 1.8 | 2.4 | 2.1 | 3.2 | 0.7 | 0.3 | <0.001 |
|  | Order | *Bacteroidales*^a^ | 39.2 | 39.3 | 40.7 | 35.5 | 45.3 | 47.6 | 44.4 | 40.6 | 29.3 | 2.0 | 4.2 | <0.001 |
|  |  | *Fibrobacterales*^a^ | 5.3 | 7.8 | 4.8 | 3.5 | 5.8 | 5.4 | 7.1 | 4.1 | 9.1 | 0.7 | 1.5 | <0.001 |
|  |  | *Clostridiales*^a^ | 45.9 | 40.8 | 47.4 | 53.6 | 40.2 | 39.8 | 38.1 | 46.5 | 50.1 | 2.1 | 4.4 | <0.001 |
|  |  | *Spirochaetales*^a^ | 0.7 | 0.9 | 0.6 | 0.7 | 1.3 | 0.9 | 1.1 | 0.8 | 2.2 | 0.2 | 0.4 | <0.001 |
|  |  | CW040^a^ | 1.7 | 1.9 | 1.0 | 1.2 | 1.4 | 1.1 | 1.4 | 1.2 | 1.4 | 0.2 | 0.5 | 0.015 |
|  |  | *Erysipelotrichales*^a^ | 1.7 | 1.9 | 1.6 | 1.8 | 1.5 | 1.6 | 1.8 | 2.5 | 1.9 | 0.3 | 0.7 | 0.181 |
|  |  | RF39 | 2.7 | 3.9 | 1.9 | 1.8 | 1.6 | 1.7 | 2.0 | 2.0 | 2.9 | 0.3 | 0.7 | <0.001 |
|  | Family | *Bacteroidales,* unknown family affiliations | 14.8 | 14.2 | 11.7 | 9.8 | 14.9 | 14.2 | 17.6 | 13.4 | 11.7 | 1.4 | 3.0 | 0.002 |
|  |  | *Bacteroidaceae* | 0.5 | 0.4 | 0.6 | 0.3 | 0.4 | 0.8 | 0.5 | 0.5 | 0.5 | 0.1 | 0.3 | 0.068 |
|  |  | *Porphyromonadaceae* | 0.7 | 1.2 | 0.6 | 0.5 | 0.9 | 0.8 | 1.2 | 0.7 | 1.2 | 0.1 | 0.2 | <0.001 |
|  |  | *Prevotellaceae* | 23.2 | 23.5 | 27.8 | 24.9 | 29.1 | 31.8 | 25.2 | 26.1 | 15.8 | 1.4 | 3.0 | <0.001 |
|  |  | *Fibrobacteraceae*^a^ | 5.3 | 7.8 | 4.8 | 3.5 | 5.8 | 5.4 | 7.1 | 4.1 | 9.1 | 0.7 | 1.5 | <0.001 |
|  |  | *Clostridiales,* unknown family affiliations | 12.2 | 10.9 | 12.0 | 11.4 | 9.9 | 9.7 | 9.1 | 12.5 | 14.5 | 0.5 | 1.2 | <0.001 |
|  |  | *Catabacteriaceae* | 1.0 | 0.9 | 0.8 | 1.1 | 1.4 | 1.1 | 1.4 | 1.0 | 0.6 | 0.2 | 0.5 | 0.054 |
|  |  | *Lachnospiraceae* | 24.0 | 22.2 | 26.4 | 32.4 | 20.1 | 19.9 | 18.4 | 23.7 | 27.7 | 1.6 | 3.4 | <0.001 |
|  |  | *Ruminococcaceae* | 7.1 | 5.9 | 6.7 | 6.9 | 7.0 | 7.3 | 7.6 | 8.0 | 6.3 | 0.7 | 1.5 | 0.216 |
|  |  | *Veillonellaceae* | 1.2 | 0.6 | 1.2 | 1.3 | 1.4 | 1.4 | 1.1 | 1.0 | 0.6 | 0.2 | 0.5 | 0.023 |
|  |  | *Spirochaetaceae*^a^ | 0.7 | 0.9 | 0.6 | 0.7 | 1.3 | 0.9 | 1.1 | 0.8 | 2.2 | 0.2 | 0.4 | <0.001 |
|  |  | F16^a^ | 1.7 | 1.9 | 1.0 | 1.2 | 1.4 | 1.1 | 1.4 | 1.2 | 1.4 | 0.2 | 0.5 | 0.015 |
|  |  | *Erysipelotrichaceae* | 1.4 | 1.3 | 1.4 | 1.7 | 1.1 | 1.3 | 1.1 | 2.3 | 1.6 | 0.3 | 0.6 | 0.022 |
|  |  | RF39, unknown family affiliations^a^ | 2.7 | 3.9 | 1.9 | 1.8 | 1.6 | 1.7 | 2.0 | 2.0 | 2.9 | 0.3 | 0.7 | <0.001 |
|  | Genus | *Bacteroidales*, unknown family and genus affiliations^a^ | 14.8 | 14.2 | 11.7 | 9.8 | 14.9 | 14.2 | 17.6 | 13.4 | 11.7 | 1.4 | 3.0 | 0.002 |
|  |  | *Bacteroides*^a^ | 0.5 | 0.4 | 0.6 | 0.3 | 0.4 | 0.8 | 0.5 | 0.5 | 0.5 | 0.1 | 0.3 | 0.068 |
|  |  | *Parabacteroides* | 0.3 | 0.3 | 0.2 | 0.2 | 0.2 | 0.3 | 0.3 | 0.3 | 0.5 | 0.1 | 0.2 | 0.127 |
|  |  | *Prevotellaceae*, unknown genus affiliations | 1.4 | 1.3 | 0.9 | 0.7 | 1.0 | 1.0 | 1.1 | 1.1 | 1.2 | 0.2 | 0.4 | 0.110 |
|  |  | *Prevotella* | 21.9 | 22.2 | 27.0 | 24.2 | 28.1 | 30.8 | 24.1 | 24.9 | 14.7 | 1.4 | 2.5 | <0.001 |
|  |  | *Fibrobacter*^a^ | 5.3 | 7.8 | 4.8 | 3.5 | 5.8 | 5.4 | 7.1 | 4.1 | 9.1 | 0.7 | 1.5 | <0.001 |
|  |  | *Clostridiales*, unknown family and genus affiliations^a^ | 12.2 | 10.9 | 12.0 | 11.4 | 9.9 | 9.7 | 9.1 | 12.5 | 14.5 | 0.5 | 1.2 | <0.001 |
|  |  | Catabacteriaceae, unknown genus affiliations^a^ | 1.0 | 0.9 | 0.8 | 1.1 | 1.4 | 1.1 | 1.4 | 1.0 | 0.6 | 0.2 | 0.5 | 0.054 |
|  |  | *Lachnospiraceae*, unknown genus affiliations | 11.3 | 11.7 | 11.4 | 13.4 | 9.9 | 10.6 | 9.0 | 10.1 | 14.2 | 0.9 | 1.8 | <0.001 |
|  |  | *Butyrivibrio* | 9.0 | 7.1 | 11.4 | 14.9 | 6.4 | 5.6 | 6.3 | 10.4 | 9.2 | 0.9 | 2.0 | <0.001 |
|  |  | *Coprococcus* | 2.1 | 1.8 | 1.8 | 2.4 | 2.2 | 2.1 | 1.6 | 1.6 | 2.1 | 0.3 | 0.6 | 0.146 |
|  |  | *Pseudobutyrivibrio* | 0.8 | 1.1 | 0.9 | 0.9 | 0.9 | 1.0 | 0.9 | 0.8 | 1.5 | 0.5 | 0.2 | 0.155 |
|  |  | *Roseburia* | 0.3 | 0.2 | 0.1 | 0.2 | 0.2 | 0.2 | 0.2 | 0.2 | 0.3 | 0.1 | 0.1 | 0.221 |
|  |  | *Ruminococcaceae*, unknown genus affiliations | 5.6 | 3.9 | 5.0 | 5.0 | 5.3 | 6.0 | 5.9 | 6.2 | 4.3 | 0.6 | 1.3 | 0.016 |
|  |  | *Ruminococcus* | 1.5 | 1.9 | 1.7 | 1.9 | 1.7 | 1.3 | 1.7 | 1.8 | 2.0 | 0.3 | 0.6 | 0.234 |
|  |  | *Veillonellaceae*, unknown genus affiliations | 1.1 | 0.5 | 1.0 | 1.1 | 1.3 | 1.3 | 1.0 | 0.9 | 0.6 | 0.2 | 0.5 | 0.017 |
|  |  | *Treponema*^a^ | 0.7 | 0.9 | 0.6 | 0.7 | 1.3 | 0.9 | 1.1 | 0.8 | 2.2 | 0.2 | 0.4 | <0.001 |
|  |  | F16, unknown genus affiliations^a^ | 1.7 | 1.9 | 1.0 | 1.2 | 1.4 | 1.1 | 1.4 | 1.2 | 1.4 | 0.2 | 0.5 | 0.015 |
|  |  | *Bulleidia* | 1.0 | 1.0 | 1.2 | 1.4 | 0.8 | 1.0 | 0.9 | 1.9 | 1.4 | 0.3 | 0.5 | 0.016 |
|  |  | *Sharpea* | 0.1 | 0.1 | 0.0 | 0.0 | 0.1 | 0.0 | 0.0 | 0.1 | 0.0 | 0.0 | 0.1 | 0.055 |
|  |  | RF39, unknown family and genus affiliations^a^ | 2.7 | 3.9 | 1.9 | 1.8 | 1.6 | 1.7 | 2.0 | 2.0 | 2.9 | 0.3 | 0.7 | <0.001 |
| Archaea | Mixed | *Methanobrevibacter gottschalkii* clade | 25.8 | 24.5 | 26.8 | 28.7 | 26.6 | 21.9 | 26.9 | 29.1 | 24.7 | 1.7 | 3.6 | 0.016 |
|  |  | *Methanobrevibacter ruminantium* clade | 65.4 | 64.9 | 63.7 | 65.2 | 62.2 | 64.3 | 61.7 | 64.9 | 65.3 | 2.3 | 4.9 | 0.694 |
|  |  | *Methanosphaera* spp. | 2.7 | 2.7 | 3.1 | 2.8 | 3.0 | 2.5 | 2.6 | 2.9 | 2.5 | 0.5 | 1.1 | 0.956 |
|  |  | ‘*Methanoplasmatales*’ | 5.9 | 7.7 | 6.3 | 3.3 | 8.3 | 11.1 | 8.5 | 3.1 | 7.5 | 1.2 | 2.6 | <0.001 |
| Ciliate protozoa | Genus | *Entodinium* | 32.5 | 45.7 | 41.2 | 36.4 | 34.1 | 35.2 | 45.6 | 34.1 | 32.2 | 2.7 | 5.7 | <0.001 |
|  |  | *Epidinium* | 1.4 | 3.0 | 2.0 | 1.7 | 1.4 | 1.8 | 1.3 | 1.4 | 0.9 | 0.4 | 0.8 | 0.001 |
|  |  | *Eremoplastron-Diploplastron* | 4.2 | 5.9 | 4.4 | 4.3 | 3.7 | 4.5 | 6.3 | 4.0 | 2.5 | 0.5 | 1.1 | <0.001 |
|  |  | *Ostracodinium* | 50.0 | 35.7 | 42.1 | 47.0 | 49.2 | 47.4 | 39.5 | 48.7 | 54.2 | 2.3 | 4.8 | <0.001 |
|  |  | *Polyplastron* | 7.6 | 6.0 | 6.5 | 6.9 | 7.4 | 7.0 | 5.1 | 7.1 | 5.4 | 1.2 | 2.5 | 0.434 |
|  |  | *Dasytricha* | 0.9 | 1.3 | 1.4 | 1.0 | 1.4 | 1.1 | 1.0 | 1.3 | 1.5 | 0.2 | 0.5 | 0.214 |
|  |  | *Isotricha* 2 | 2.2 | 1.2 | 1.4 | 1.8 | 1.6 | 1.9 | 0.7 | 2.3 | 2.1 | 0.3 | 0.6 | <0.001 |
| Fungi | Sub-genus | *Caecomyces* 1 | 59.8 | 66.0 | 67.5 | 72.0 | 62.7 | 70.1 | 70.4 | 65.4 | 62.5 | 5.5 | 11.5 | 0.388 |
|  |  | KF1 | 1.9 | 2.3 | 2.3 | 3.2 | 3.7 | 2.5 | 0.7 | 2.2 | 2.9 | 1.2 | 2.4 | 0.432 |
|  |  | *Neocallimastix* 1 | 16.3 | 11.7 | 10.6 | 11.3 | 13.5 | 8.2 | 10.4 | 14.6 | 15.4 | 3.6 | 7.5 | 0.395 |
|  |  | *Orpinomyces* 5 | 1.0 | 1.2 | 1.9 | 0.8 | 0.9 | 0.5 | 2.5 | 0.6 | 0.4 | 1.0 | 2.1 | 0.501 |
|  |  | *Orpinomyces* 6 | 0.0 | 0.7 | 0.7 | 1.9 | 1.0 | 1.8 | 2.9 | 1.2 | 0.8 | 0.6 | 1.3 | 0.012 |
|  |  | *Piromyces* 2 | 13.9 | 12.8 | 9.4 | 5.9 | 12.0 | 12.7 | 8.2 | 11.6 | 9.4 | 2.3 | 4.8 | 0.043 |
|  |  | *Piromyces* 7 | 3.1 | 2.4 | 2.8 | 2.0 | 2.6 | 1.8 | 3.6 | 1.8 | 2.7 | 1.4 | 2.9 | 0.920 |
|  |  | SK3 | 4.1 | 2.6 | 4.6 | 2.7 | 3.6 | 2.5 | 1.4 | 2.4 | 5.7 | 1.1 | 2.4 | 0.039 |

^a^Value similar to that of the next highest taxonomic rank that contains that group.

**B. Pasture-fed sheep**

| Microbial group | Taxonomic rank | Taxon | DNA extraction method | | | | | | | | | | | |
| --- | --- | --- | --- | --- | --- | --- | --- | --- | --- | --- | --- | --- | --- | --- |
|  |  |  | PCBB | PCFI | PCQI | PCSA | PSP1 | PSP2 | QIAG | RBBC | ZYMO | SE | LSD | *p* |
| Bacteria | Phylum | *Bacteroidetes* | 63.0 | 65.9 | 63.2 | 58.6 | 75.0 | 74.7 | 72.5 | 63.9 | 55.8 | 1.6 | 3.4 | <0.001 |
|  |  | *Fibrobacteres* | 2.6 | 4.0 | 1.8 | 1.5 | 2.4 | 2.5 | 3.1 | 1.7 | 7.2 | 0.3 | 0.6 | <0.001 |
|  |  | *Firmicutes* | 28.2 | 25.8 | 27.4 | 29.4 | 19.0 | 19.6 | 21.4 | 25.9 | 30.2 | 1.3 | 2.8 | <0.001 |
|  |  | *Spirochaetes* | 0.5 | 0.8 | 0.4 | 0.4 | 0.7 | 0.6 | 0.9 | 0.5 | 1.2 | 0.2 | 0.3 | 0.001 |
|  |  | TM7 | 0.3 | 0.3 | 0.2 | 0.2 | 0.2 | 0.1 | 0.1 | 0.2 | 0.2 | 0.1 | 0.1 | 0.012 |
|  |  | *Tenericutes* | 5.2 | 3.1 | 6.7 | 9.7 | 2.7 | 2.3 | 2.0 | 7.6 | 5.3 | 0.5 | 1.0 | <0.001 |
|  | Class | *Bacteroidia*^a^ | 63.0 | 65.9 | 63.2 | 58.6 | 75.0 | 74.7 | 72.5 | 63.9 | 55.8 | 1.6 | 3.4 | <0.001 |
|  |  | *Fibrobacteres*^a^ | 2.6 | 4.0 | 1.8 | 1.5 | 2.4 | 2.5 | 3.1 | 1.7 | 7.2 | 0.3 | 0.6 | <0.001 |
|  |  | *Clostridia*^a^ | 28.2 | 25.8 | 27.4 | 29.4 | 19.0 | 19.6 | 21.4 | 25.9 | 30.2 | 1.3 | 2.8 | <0.001 |
|  |  | *Spirochaetes*^a^ | 0.5 | 0.8 | 0.4 | 0.4 | 0.7 | 0.6 | 0.9 | 0.5 | 1.2 | 0.2 | 0.3 | 0.001 |
|  |  | TM7-3^a^ | 0.3 | 0.3 | 0.2 | 0.2 | 0.2 | 0.1 | 0.1 | 0.2 | 0.2 | 0.1 | 0.1 | 0.012 |
|  |  | *Erysipelotrichi* | 4.9 | 2.7 | 6.5 | 9.5 | 2.4 | 2.1 | 1.7 | 7.3 | 4.7 | 0.5 | 1.0 | <0.001 |
|  |  | *Mollicutes* | 0.4 | 0.4 | 0.2 | 0.2 | 0.3 | 0.2 | 0.3 | 0.3 | 0.5 | 0.1 | 0.2 | 0.007 |
|  | Order | *Bacteroidales*^a^ | 63.0 | 65.9 | 63.2 | 58.6 | 75.0 | 74.7 | 72.5 | 63.9 | 55.8 | 1.6 | 3.4 | <0.001 |
|  |  | *Fibrobacterales*^a^ | 2.6 | 4.0 | 1.8 | 1.5 | 2.4 | 2.5 | 3.1 | 1.7 | 7.2 | 0.3 | 0.6 | <0.001 |
|  |  | *Clostridiales*^a^ | 2.6 | 4.0 | 1.8 | 1.5 | 2.4 | 2.5 | 3.1 | 1.7 | 7.2 | 0.3 | 0.6 | <0.001 |
|  |  | *Spirochaetales*^a^ | 0.5 | 0.8 | 0.4 | 0.4 | 0.7 | 0.6 | 0.9 | 0.5 | 1.2 | 0.2 | 0.3 | 0.001 |
|  |  | CW040^a^ | 0.3 | 0.3 | 0.2 | 0.2 | 0.2 | 0.1 | 0.1 | 0.2 | 0.2 | 0.1 | 0.1 | 0.012 |
|  |  | *Erysipelotrichales*^a^ | 4.9 | 2.7 | 6.5 | 9.5 | 2.4 | 2.1 | 1.7 | 7.3 | 4.7 | 0.5 | 1.0 | <0.001 |
|  |  | RF39 | 0.1 | 0.2 | 0.1 | 0.1 | 0.1 | 0.1 | 0.1 | 0.2 | 0.2 | 0.5 | 0.1 | 0.105 |
|  | Family | *Bacteroidales,* unknown family affiliations | 3.8 | 3.3 | 5.3 | 4.4 | 6.4 | 5.5 | 5.9 | 5.1 | 3.8 | 0.7 | 1.4 | 0.003 |
|  |  | *Bacteroidaceae* | 0.8 | 1.3 | 0.7 | 0.5 | 1.0 | 1.1 | 1.1 | 0.7 | 1.6 | 0.1 | 0.3 | <0.001 |
|  |  | *Porphyromonadaceae* | 1.7 | 2.3 | 1.1 | 1.0 | 1.4 | 1.4 | 1.6 | 1.1 | 2.6 | 0.2 | 0.5 | <0.001 |
|  |  | *Prevotellaceae* | 56.6 | 59.0 | 56.2 | 52.8 | 66.2 | 66.7 | 63.9 | 56.9 | 47.8 | 1.9 | 4.0 | <0.001 |
|  |  | *Fibrobacteraceae*^a^ | 2.6 | 4.0 | 1.8 | 1.5 | 2.4 | 2.5 | 3.1 | 1.7 | 7.2 | 0.3 | 0.6 | <0.001 |
|  |  | *Clostridiales,* unknown family affiliations | 3.7 | 4.1 | 3.0 | 3.3 | 2.3 | 2.2 | 2.6 | 3.0 | 4.6 | 0.3 | 0.7 | <0.001 |
|  |  | *Catabacteriaceae* | 0.0 | 0.0 | 0.0 | 0.0 | 0.0 | 0.0 | 0.0 | 0.0 | 0.0 | 0.0 | 0.0 | 0.174 |
|  |  | *Lachnospiraceae* | 19.1 | 17.1 | 19.8 | 21.1 | 13.2 | 14.2 | 14.9 | 18.1 | 20.8 | 1.2 | 2.5 | <0.001 |
|  |  | *Ruminococcaceae* | 2.3 | 1.9 | 2.1 | 2.4 | 1.5 | 1.3 | 1.6 | 2.4 | 2.6 | 0.2 | 0.5 | <0.001 |
|  |  | *Veillonellaceae* | 2.5 | 2.5 | 2.3 | 2.0 | 1.8 | 1.7 | 2.1 | 2.2 | 1.8 | 0.4 | 0.8 | 0.305 |
|  |  | *Spirochaetaceae*^a^ | 0.5 | 0.8 | 0.4 | 0.4 | 0.7 | 0.6 | 0.9 | 0.5 | 1.2 | 0.2 | 0.3 | 0.001 |
|  |  | F16^a^ | 0.3 | 0.3 | 0.2 | 0.2 | 0.2 | 0.1 | 0.1 | 0.2 | 0.2 | 0.1 | 0.1 | 0.012 |
|  |  | *Erysipelotrichaceae* | 4.9 | 2.6 | 6.5 | 9.5 | 2.4 | 2.1 | 1.6 | 7.3 | 4.6 | 0.5 | 0.9 | <0.01 |
|  |  | RF39, unknown family affiliations^a^ | 0.1 | 0.2 | 0.1 | 0.1 | 0.1 | 0.1 | 0.1 | 0.2 | 0.2 | 0.5 | 0.1 | 0.105 |
|  | Genus | *Bacteroidales*, unknown family and genus affiliations^a^ | 3.8 | 3.3 | 5.3 | 4.4 | 6.4 | 5.5 | 5.9 | 5.1 | 3.8 | 0.7 | 1.4 | 0.003 |
|  |  | *Bacteroides*^a^ | 0.8 | 1.3 | 0.7 | 0.5 | 1.0 | 1.1 | 1.1 | 0.7 | 1.6 | 0.1 | 0.3 | <0.001 |
|  |  | *Parabacteroides* | 1.7 | 2.3 | 1.1 | 1.0 | 1.4 | 1.4 | 1.5 | 1.1 | 2.6 | 0.2 | 0.5 | <0.001 |
|  |  | *Prevotellaceae*, unknown genus affiliations | 0.7 | 0.7 | 0.6 | 0.4 | 0.5 | 0.7 | 0.8 | 0.6 | 0.7 | 0.1 | 0.3 | 0.357 |
|  |  | *Prevotella* | 55.9 | 58.3 | 55.6 | 52.3 | 65.7 | 66.0 | 63.1 | 56.3 | 47.1 | 1.9 | 4.0 | <0.001 |
|  |  | *Fibrobacter*^a^ | 2.6 | 4.0 | 1.8 | 1.5 | 2.4 | 2.5 | 3.1 | 1.7 | 7.2 | 0.3 | 0.6 | <0.001 |
|  |  | *Clostridiales*, unknown family and genus affiliations^a^ | 3.7 | 4.1 | 3.0 | 3.3 | 2.3 | 2.2 | 2.6 | 3.0 | 4.6 | 0.3 | 0.7 | <0.001 |
|  |  | Catabacteriaceae, unknown genus affiliations^a^ | 0.0 | 0.0 | 0.0 | 0.0 | 0.0 | 0.0 | 0.0 | 0.0 | 0.0 | 0.0 | 0.0 | 0.174 |
|  |  | *Lachnospiraceae*, unknown genus affiliations | 8.2 | 8.2 | 8.7 | 8.5 | 5.8 | 6.6 | 7.1 | 7.6 | 9.8 | 0.6 | 1.4 | <0.001 |
|  |  | *Butyrivibrio* | 1.7 | 1.3 | 2.3 | 3.0 | 1.4 | 1.4 | 1.4 | 2.3 | 2.1 | 0.2 | 0.5 | <0.001 |
|  |  | *Coprococcus* | 3.4 | 2.7 | 3.2 | 3.1 | 1.9 | 2.0 | 1.8 | 2.8 | 3.0 | 0.3 | 0.6 | <0.001 |
|  |  | *Pseudobutyrivibrio* | 2.6 | 2.6 | 2.6 | 2.4 | 2.1 | 2.1 | 2.7 | 2.3 | 2.7 | 0.2 | 0.5 | 0.099 |
|  |  | *Roseburia* | 1.2 | 0.9 | 1.0 | 1.3 | 0.8 | 0.9 | 0.8 | 1.2 | 1.0 | 0.2 | 0.4 | 0.048 |
|  |  | *Ruminococcaceae*, unknown genus affiliations | 1.3 | 1.0 | 1.3 | 1.3 | 0.9 | 0.8 | 1.0 | 1.2 | 1.5 | 0.1 | 0.3 | 0.003 |
|  |  | *Ruminococcus* | 1.0 | 0.8 | 0.7 | 1.1 | 0.6 | 0.5 | 0.5 | 1.1 | 1.0 | 0.2 | 0.3 | 0.002 |
|  |  | *Veillonellaceae*, unknown genus affiliations | 2.0 | 1.9 | 1.7 | 1.6 | 1.4 | 1.4 | 1.6 | 1.6 | 1.3 | 0.3 | 0.6 | 0.460 |
|  |  | *Treponema*^a^ | 0.5 | 0.8 | 0.4 | 0.4 | 0.7 | 0.6 | 0.9 | 0.5 | 1.2 | 0.2 | 0.3 | 0.001 |
|  |  | F16, unknown genus affiliations^a^ | 0.3 | 0.3 | 0.2 | 0.2 | 0.2 | 0.1 | 0.1 | 0.2 | 0.2 | 0.1 | 0.1 | 0.012 |
|  |  | *Bulleidia* | 0.4 | 0.3 | 1.1 | 0.9 | 0.3 | 0.4 | 0.3 | 0.6 | 0.9 | 0.1 | 0.2 | <0.001 |
|  |  | *Sharpea* | 4.4 | 2.3 | 5.4 | 8.5 | 2.0 | 1.7 | 1.3 | 6.7 | 3.7 | 0.5 | 1.0 | <0.001 |
|  |  | RF39, unknown family and genus affiliations^a^ | 0.1 | 0.2 | 0.1 | 0.1 | 0.1 | 0.1 | 0.1 | 0.2 | 0.2 | 0.5 | 0.1 | 0.105 |
| Archaea | Mixed | *Methanobrevibacter gottschalkii* clade | 9.0 | 10.6 | 11.1 | 9.0 | 9.2 | 8.2 | 10.5 | 9.2 | 9.3 | 0.8 | 1.8 | 0.052 |
|  |  | *Methanobrevibacter ruminantium* clade | 62.9 | 58.1 | 56.5 | 60.8 | 57.4 | 61.8 | 57.9 | 64.0 | 63.9 | 1.6 | 3.4 | <0.001 |
|  |  | *Methanosphaera* spp. | 24.0 | 21.1 | 27.0 | 28.1 | 21.7 | 20.3 | 18.3 | 24.0 | 23.8 | 1.1 | 2.4 | <0.001 |
|  |  | ‘*Methanoplasmatales*’ | 4.0 | 10.2 | 5.5 | 2.0 | 11.4 | 9.6 | 13.2 | 2.7 | 3.0 | 1.1 | 2.2 | <0.002 |

^a^Value similar to that of the next highest taxonomic rank that contains that group.
