# Supplementary material for: Effect of DNA Extraction Methods and Sampling Techniques on the Apparent Structure of Cow and Sheep Rumen Microbial Communities
Source: PLoS One. 2013 Sep 11;8(9):e74787. doi: 10.1371/journal.pone.0074787 (PMC3770609; doi:10.1371/journal.pone.0074787)
Supplement: Table S4 — Correlations of apparent microbial community structures following extraction of DNA with different methods. Pearson (A, B) and Spearman’s rank (C, D) correlations between different parts of the microbial community at different taxonomic levels were calculated using mean abundances of dominant bacterial, archaeal, fungal and ciliate protozoal taxa (from data in Table S3) measured in DNA from (A, C) cow and (B, D) sheep rumen content samples extracted using nine different extraction methods (Table 1). (DOCX) [file pone.0074787.s005.docx]

**Table S4. Correlations of apparent microbial community structures following extraction of DNA with different methods.**

Pearson (A, B) and Spearman’s rank (C, D) correlations between different parts of the microbial community at different taxonomic levels were calculated using mean abundances of dominant bacterial, archaeal, fungal and ciliate protozoal taxa (from data in Table S3) measured in DNA from (A, C) cow and (B, D) sheep rumen content samples extracted using nine different extraction methods (Table 1).

**A. Pearson, cow**

| Microbial group | Taxonomic rank | DNA extraction method | DNA extraction method | | | | | | | |
| --- | --- | --- | --- | --- | --- | --- | --- | --- | --- | --- |
|  |  |  | RCBB | PCFI | PCQI | PCSA | PSP1 | PSP2 | QIAG | RBBC |
| Bacteria | Phylum | PCFI | 0.004 |  |  |  |  |  |  |  |
|  |  | PCQI | 0.000 | 0.005 |  |  |  |  |  |  |
|  |  | PCSA | 0.012 | 0.030 | 0.013 |  |  |  |  |  |
|  |  | PSP1 | 0.017 | 0.008 | 0.016 | 0.057 |  |  |  |  |
|  |  | PSP2 | 0.024 | 0.012 | 0.023 | 0.069 | 0.001 |  |  |  |
|  |  | QIAG | 0.022 | 0.009 | 0.021 | 0.066 | 0.001 | 0.001 |  |  |
|  |  | RBBC | 0.000 | 0.005 | 0.000 | 0.014 | 0.015 | 0.022 | 0.020 |  |
|  |  | ZYMO | 0.032 | 0.052 | 0.033 | 0.009 | 0.091 | 0.107 | 0.099 | 0.037 |
|  | Family | PCFI | 0.008 |  |  |  |  |  |  |  |
|  |  | PCQI | 0.012 | 0.017 |  |  |  |  |  |  |
|  |  | PCSA | 0.033 | 0.050 | 0.020 |  |  |  |  |  |
|  |  | PSP1 | 0.029 | 0.023 | 0.024 | 0.082 |  |  |  |  |
|  |  | PSP2 | 0.044 | 0.036 | 0.031 | 0.094 | 0.003 |  |  |  |
|  |  | QIAG | 0.032 | 0.023 | 0.049 | 0.110 | 0.012 | 0.022 |  |  |
|  |  | RBBC | 0.006 | 0.016 | 0.005 | 0.033 | 0.019 | 0.027 | 0.034 |  |
|  |  | ZYMO | 0.057 | 0.067 | 0.077 | 0.052 | 0.149 | 0.177 | 0.145 | 0.083 |
|  | Genus | PCFI | 0.013 |  |  |  |  |  |  |  |
|  |  | PCQI | 0.021 | 0.033 |  |  |  |  |  |  |
|  |  | PCSA | 0.047 | 0.071 | 0.017 |  |  |  |  |  |
|  |  | PSP1 | 0.028 | 0.025 | 0.025 | 0.078 |  |  |  |  |
|  |  | PSP2 | 0.042 | 0.037 | 0.030 | 0.085 | 0.003 |  |  |  |
|  |  | QIAG | 0.025 | 0.021 | 0.051 | 0.108 | 0.014 | 0.026 |  |  |
|  |  | RBBC | 0.010 | 0.029 | 0.006 | 0.030 | 0.023 | 0.030 | 0.037 |  |
|  |  | ZYMO | 0.068 | 0.069 | 0.109 | 0.108 | 0.150 | 0.175 | 0.134 | 0.105 |
| Archaea | Mixed | PCFI | 0.001 |  |  |  |  |  |  |  |
|  |  | PCQI | 0.000 | 0.002 |  |  |  |  |  |  |
|  |  | PCSA | 0.003 | 0.007 | 0.002 |  |  |  |  |  |
|  |  | PSP1 | 0.001 | 0.001 | 0.001 | 0.004 |  |  |  |  |
|  |  | PSP2 | 0.008 | 0.004 | 0.010 | 0.020 | 0.007 |  |  |  |
|  |  | QIAG | 0.001 | 0.002 | 0.001 | 0.004 | 0.000 | 0.008 |  |  |
|  |  | RBBC | 0.004 | 0.008 | 0.002 | 0.000 | 0.004 | 0.022 | 0.005 |  |
|  |  | ZYMO | 0.001 | 0.000 | 0.002 | 0.006 | 0.001 | 0.004 | 0.002 | 0.007 |
| Ciliate protozoa | Genus | PCFI | 0.090 |  |  |  |  |  |  |  |
|  |  | PCQI | 0.032 | 0.015 |  |  |  |  |  |  |
|  |  | PCSA | 0.006 | 0.052 | 0.011 |  |  |  |  |  |
|  |  | PSP1 | 0.001 | 0.075 | 0.023 | 0.002 |  |  |  |  |
|  |  | PSP2 | 0.003 | 0.060 | 0.015 | 0.000 | 0.001 |  |  |  |
|  |  | QIAG | 0.065 | 0.003 | 0.007 | 0.033 | 0.052 | 0.040 |  |  |
|  |  | RBBC | 0.001 | 0.073 | 0.022 | 0.002 | 0.000 | 0.001 | 0.050 |  |
|  |  | ZYMO | 0.002 | 0.110 | 0.045 | 0.012 | 0.004 | 0.009 | 0.082 | 0.005 |
| Fungi | Sub-genus | PCFI | 0.005 |  |  |  |  |  |  |  |
|  |  | PCQI | 0.011 | 0.003 |  |  |  |  |  |  |
|  |  | PCSA | 0.018 | 0.007 | 0.002 |  |  |  |  |  |
|  |  | PSP1 | 0.003 | 0.001 | 0.004 | 0.007 |  |  |  |  |
|  |  | PSP2 | 0.014 | 0.002 | 0.003 | 0.007 | 0.005 |  |  |  |
|  |  | QIAG | 0.017 | 0.005 | 0.003 | 0.002 | 0.007 | 0.005 |  |  |
|  |  | RBBC | 0.003 | 0.002 | 0.004 | 0.007 | 0.001 | 0.006 | 0.007 |  |
|  |  | ZYMO | 0.005 | 0.005 | 0.005 | 0.006 | 0.002 | 0.011 | 0.010 | 0.002 |

**B. Pearson, sheep**

| Microbial group | Taxonomic rank | DNA extraction method | DNA extraction method | | | | | | | |
| --- | --- | --- | --- | --- | --- | --- | --- | --- | --- | --- |
|  |  |  | RCBB | PCFI | PCQI | PCSA | PSP1 | PSP2 | QIAG | RBBC |
| Bacteria | Phylum | PCFI | 0.002 |  |  |  |  |  |  |  |
|  |  | PCQI | 0.001 | 0.004 |  |  |  |  |  |  |
|  |  | PCSA | 0.005 | 0.013 | 0.003 |  |  |  |  |  |
|  |  | PSP1 | 0.019 | 0.009 | 0.017 | 0.035 |  |  |  |  |
|  |  | PSP2 | 0.017 | 0.008 | 0.016 | 0.034 | 0.000 |  |  |  |
|  |  | QIAG | 0.012 | 0.004 | 0.012 | 0.028 | 0.001 | 0.001 |  |  |
|  |  | RBBC | 0.002 | 0.004 | 0.000 | 0.005 | 0.014 | 0.013 | 0.010 |  |
|  |  | ZYMO | 0.006 | 0.011 | 0.009 | 0.010 | 0.039 | 0.036 | 0.028 | 0.013 |
|  | Family | PCFI | 0.002 |  |  |  |  |  |  |  |
|  |  | PCQI | 0.001 | 0.006 |  |  |  |  |  |  |
|  |  | PCSA | 0.006 | 0.016 | 0.003 |  |  |  |  |  |
|  |  | PSP1 | 0.011 | 0.006 | 0.014 | 0.028 |  |  |  |  |
|  |  | PSP2 | 0.009 | 0.004 | 0.012 | 0.025 | 0.000 |  |  |  |
|  |  | QIAG | 0.007 | 0.003 | 0.011 | 0.024 | 0.001 | 0.000 |  |  |
|  |  | RBBC | 0.002 | 0.006 | 0.001 | 0.004 | 0.010 | 0.009 | 0.009 |  |
|  |  | ZYMO | 0.008 | 0.012 | 0.010 | 0.013 | 0.030 | 0.027 | 0.023 | 0.014 |
|  | Genus | PCFI | 0.001 |  |  |  |  |  |  |  |
|  |  | PCQI | 0.001 | 0.004 |  |  |  |  |  |  |
|  |  | PCSA | 0.004 | 0.011 | 0.002 |  |  |  |  |  |
|  |  | PSP1 | 0.004 | 0.003 | 0.004 | 0.012 |  |  |  |  |
|  |  | PSP2 | 0.003 | 0.002 | 0.004 | 0.012 | 0.000 |  |  |  |
|  |  | QIAG | 0.003 | 0.002 | 0.004 | 0.013 | 0.000 | 0.000 |  |  |
|  |  | RBBC | 0.002 | 0.005 | 0.001 | 0.002 | 0.005 | 0.005 | 0.006 |  |
|  |  | ZYMO | 0.007 | 0.005 | 0.010 | 0.014 | 0.014 | 0.012 | 0.010 | 0.012 |
| Archaea | Mixed | PCFI | 0.007 |  |  |  |  |  |  |  |
|  |  | PCQI | 0.004 | 0.019 |  |  |  |  |  |  |
|  |  | PCSA | 0.006 | 0.024 | 0.000 |  |  |  |  |  |
|  |  | PSP1 | 0.009 | 0.001 | 0.022 | 0.026 |  |  |  |  |
|  |  | PSP2 | 0.010 | 0.000 | 0.024 | 0.029 | 0.001 |  |  |  |
|  |  | QIAG | 0.024 | 0.005 | 0.045 | 0.052 | 0.006 | 0.003 |  |  |
|  |  | RBBC | 0.000 | 0.008 | 0.003 | 0.005 | 0.012 | 0.012 | 0.027 |  |
|  |  | ZYMO | 0.000 | 0.007 | 0.004 | 0.006 | 0.011 | 0.011 | 0.025 | 0.000 |

**C. Spearman, cow**

| Microbial group | Taxonomic rank | DNA extraction method | DNA extraction method | | | | | | | |
| --- | --- | --- | --- | --- | --- | --- | --- | --- | --- | --- |
|  |  |  | RCBB | PCFI | PCQI | PCSA | PSP1 | PSP2 | QIAG | RBBC |
| Bacteria | Phylum | PCFI | 0.000 |  |  |  |  |  |  |  |
|  |  | PCQI | 0.000 | 0.000 |  |  |  |  |  |  |
|  |  | PCSA | 0.057 | 0.057 | 0.057 |  |  |  |  |  |
|  |  | PSP1 | 0.114 | 0.114 | 0.114 | 0.171 |  |  |  |  |
|  |  | PSP2 | 0.057 | 0.057 | 0.057 | 0.114 | 0.057 |  |  |  |
|  |  | QIAG | 0.057 | 0.057 | 0.057 | 0.114 | 0.057 | 0.000 |  |  |
|  |  | RBBC | 0.057 | 0.057 | 0.057 | 0.000 | 0.171 | 0.114 | 0.114 |  |
|  |  | ZYMO | 0.057 | 0.057 | 0.057 | 0.114 | 0.057 | 0.114 | 0.114 | 0.114 |
|  | Family | PCFI | 0.053 |  |  |  |  |  |  |  |
|  |  | PCQI | 0.022 | 0.079 |  |  |  |  |  |  |
|  |  | PCSA | 0.018 | 0.084 | 0.004 |  |  |  |  |  |
|  |  | PSP1 | 0.057 | 0.123 | 0.062 | 0.066 |  |  |  |  |
|  |  | PSP2 | 0.035 | 0.110 | 0.013 | 0.018 | 0.035 |  |  |  |
|  |  | QIAG | 0.066 | 0.062 | 0.088 | 0.092 | 0.053 | 0.084 |  |  |
|  |  | RBBC | 0.022 | 0.057 | 0.022 | 0.026 | 0.079 | 0.040 | 0.070 |  |
|  |  | ZYMO | 0.079 | 0.044 | 0.084 | 0.079 | 0.136 | 0.114 | 0.136 | 0.092 |
|  | Genus | PCFI | 0.031 |  |  |  |  |  |  |  |
|  |  | PCQI | 0.026 | 0.052 |  |  |  |  |  |  |
|  |  | PCSA | 0.039 | 0.066 | 0.021 |  |  |  |  |  |
|  |  | PSP1 | 0.040 | 0.057 | 0.061 | 0.051 |  |  |  |  |
|  |  | PSP2 | 0.029 | 0.077 | 0.032 | 0.038 | 0.026 |  |  |  |
|  |  | QIAG | 0.036 | 0.036 | 0.066 | 0.071 | 0.013 | 0.044 |  |  |
|  |  | RBBC | 0.029 | 0.043 | 0.021 | 0.034 | 0.073 | 0.052 | 0.061 |  |
|  |  | ZYMO | 0.084 | 0.052 | 0.065 | 0.084 | 0.082 | 0.105 | 0.074 | 0.083 |
| Archaea | Mixed | PCFI | 0.001 |  |  |  |  |  |  |  |
|  |  | PCQI | 0.000 | 0.002 |  |  |  |  |  |  |
|  |  | PCSA | 0.003 | 0.007 | 0.002 |  |  |  |  |  |
|  |  | PSP1 | 0.001 | 0.001 | 0.001 | 0.004 |  |  |  |  |
|  |  | PSP2 | 0.008 | 0.004 | 0.010 | 0.020 | 0.007 |  |  |  |
|  |  | QIAG | 0.001 | 0.002 | 0.001 | 0.004 | 0.000 | 0.008 |  |  |
|  |  | RBBC | 0.004 | 0.008 | 0.002 | 0.000 | 0.004 | 0.022 | 0.005 |  |
|  |  | ZYMO | 0.001 | 0.000 | 0.002 | 0.006 | 0.001 | 0.004 | 0.002 | 0.007 |
| Ciliate protozoa | Genus | PCFI | 0.143 |  |  |  |  |  |  |  |
|  |  | PCQI | 0.107 | 0.036 |  |  |  |  |  |  |
|  |  | PCSA | 0.000 | 0.143 | 0.107 |  |  |  |  |  |
|  |  | PSP1 | 0.036 | 0.179 | 0.143 | 0.036 |  |  |  |  |
|  |  | PSP2 | 0.000 | 0.143 | 0.107 | 0.000 | 0.036 |  |  |  |
|  |  | QIAG | 0.179 | 0.036 | 0.071 | 0.179 | 0.214 | 0.179 |  |  |
|  |  | RBBC | 0.000 | 0.143 | 0.107 | 0.000 | 0.036 | 0.000 | 0.179 |  |
|  |  | ZYMO | 0.036 | 0.179 | 0.143 | 0.036 | 0.000 | 0.036 | 0.214 | 0.036 |
| Fungi | Sub-genus | PCFI | 0.024 |  |  |  |  |  |  |  |
|  |  | PCQI | 0.000 | 0.024 |  |  |  |  |  |  |
|  |  | PCSA | 0.095 | 0.119 | 0.095 |  |  |  |  |  |
|  |  | PSP1 | 0.095 | 0.119 | 0.095 | 0.000 |  |  |  |  |
|  |  | PSP2 | 0.071 | 0.048 | 0.071 | 0.048 | 0.048 |  |  |  |
|  |  | QIAG | 0.286 | 0.310 | 0.286 | 0.381 | 0.381 | 0.381 |  |  |
|  |  | RBBC | 0.048 | 0.071 | 0.048 | 0.024 | 0.024 | 0.024 | 0.357 |  |
|  |  | ZYMO | 0.048 | 0.071 | 0.048 | 0.024 | 0.024 | 0.024 | 0.357 | 0.000 |

**D. Spearman, sheep**

| Microbial group | Taxonomic rank | DNA extraction method | DNA extraction method | | | | | | | |
| --- | --- | --- | --- | --- | --- | --- | --- | --- | --- | --- |
|  |  |  | RCBB | PCFI | PCQI | PCSA | PSP1 | PSP2 | QIAG | RBBC |
| Bacteria | Phylum | PCFI | 0.057 |  |  |  |  |  |  |  |
|  |  | PCQI | 0.000 | 0.057 |  |  |  |  |  |  |
|  |  | PCSA | 0.000 | 0.057 | 0.000 |  |  |  |  |  |
|  |  | PSP1 | 0.000 | 0.057 | 0.000 | 0.000 |  |  |  |  |
|  |  | PSP2 | 0.057 | 0.000 | 0.057 | 0.057 | 0.057 |  |  |  |
|  |  | QIAG | 0.057 | 0.000 | 0.057 | 0.057 | 0.057 | 0.000 |  |  |
|  |  | RBBC | 0.000 | 0.057 | 0.000 | 0.000 | 0.000 | 0.057 | 0.057 |  |
|  |  | ZYMO | 0.057 | 0.000 | 0.057 | 0.057 | 0.057 | 0.000 | 0.000 | 0.057 |
|  | Family | PCFI | 0.044 |  |  |  |  |  |  |  |
|  |  | PCQI | 0.013 | 0.079 |  |  |  |  |  |  |
|  |  | PCSA | 0.018 | 0.088 | 0.004 |  |  |  |  |  |
|  |  | PSP1 | 0.009 | 0.044 | 0.031 | 0.035 |  |  |  |  |
|  |  | PSP2 | 0.035 | 0.018 | 0.070 | 0.079 | 0.018 |  |  |  |
|  |  | QIAG | 0.070 | 0.040 | 0.092 | 0.097 | 0.044 | 0.022 |  |  |
|  |  | RBBC | 0.022 | 0.092 | 0.009 | 0.004 | 0.040 | 0.084 | 0.101 |  |
|  |  | ZYMO | 0.044 | 0.040 | 0.088 | 0.079 | 0.044 | 0.048 | 0.079 | 0.084 |
|  | Genus | PCFI | 0.055 |  |  |  |  |  |  |  |
|  |  | PCQI | 0.026 | 0.103 |  |  |  |  |  |  |
|  |  | PCSA | 0.026 | 0.118 | 0.018 |  |  |  |  |  |
|  |  | PSP1 | 0.040 | 0.025 | 0.077 | 0.088 |  |  |  |  |
|  |  | PSP2 | 0.049 | 0.017 | 0.087 | 0.105 | 0.013 |  |  |  |
|  |  | QIAG | 0.081 | 0.026 | 0.118 | 0.144 | 0.019 | 0.019 |  |  |
|  |  | RBBC | 0.019 | 0.101 | 0.027 | 0.006 | 0.074 | 0.086 | 0.130 |  |
|  |  | ZYMO | 0.064 | 0.026 | 0.091 | 0.121 | 0.031 | 0.032 | 0.052 | 0.103 |
| Archaea | Mixed | PCFI | 0.007 |  |  |  |  |  |  |  |
|  |  | PCQI | 0.004 | 0.019 |  |  |  |  |  |  |
|  |  | PCSA | 0.006 | 0.024 | 0.000 |  |  |  |  |  |
|  |  | PSP1 | 0.009 | 0.001 | 0.022 | 0.026 |  |  |  |  |
|  |  | PSP2 | 0.010 | 0.000 | 0.024 | 0.029 | 0.001 |  |  |  |
|  |  | QIAG | 0.024 | 0.005 | 0.045 | 0.052 | 0.006 | 0.003 |  |  |
|  |  | RBBC | 0.000 | 0.008 | 0.003 | 0.005 | 0.012 | 0.012 | 0.027 |  |
|  |  | ZYMO | 0.000 | 0.007 | 0.004 | 0.006 | 0.011 | 0.011 | 0.025 | 0.000 |
