# Supplementary material for: Effect of DNA Extraction Methods and Sampling Techniques on the Apparent Structure of Cow and Sheep Rumen Microbial Communities
Source: PLoS One. 2013 Sep 11;8(9):e74787. doi: 10.1371/journal.pone.0074787 (PMC3770609; doi:10.1371/journal.pone.0074787)
Supplement: Table S5 — Effect of DNA extraction method on the apparent rumen microbial community structure post-hoc values. DNA extraction methods (Table 1) that do not share a letter are significantly different for the particular taxon in which the letters are listed (p < 0.05, ANOVA, Scheffe post-hoc test). Test was performed on the individual replicates that underlie the data in Table S3. (DOCX) [file pone.0074787.s006.docx]

**Table S5. Effect of DNA extraction method on the apparent rumen microbial community structure *post-hoc* values.**

DNA extraction methods (Table 1) that do not share a letter are significantly different for the particular taxon in which the letters are listed (*p* < 0.05, ANOVA, Scheffe *post-hoc* test). Test was performed on the individual replicates that underlie the data in Table S3.

**A. Hay-fed cow**

| Microbial group | Taxonomic rank | Taxon | DNA extraction method | | | | | | | | |
| --- | --- | --- | --- | --- | --- | --- | --- | --- | --- | --- | --- |
|  |  |  | PCBB | PCFI | PCQI | PCSA | PSP1 | PSP2 | QIAG | RBBC | ZYMO |
| Bacteria | Phylum | *Bacteroidetes* | bc | bc | bc | ab | c | c | bc | bc | a |
|  |  | *Fibrobacteres* | abc | cd | abc | a | abc | abc | bcd | ab | d |
|  |  | *Firmicutes* | ab | a | ab | b | a | a | a | ab | b |
|  |  | *Spirochaetes* | a | a | a | a | ab | a | a | a | b |
|  |  | TM7 | a | a | a | a | a | a | a | a | a |
|  |  | *Tenericutes* | ab | b | a | a | a | a | ab | ab | ab |
|  | Class | *Bacteroidia* | bc | bc | bc | ab | c | c | bc | bc | a |
|  |  | *Fibrobacteres* | abc | cd | abc | a | abc | abc | bcd | ab | d |
|  |  | *Clostridia* | ab | a | ab | b | a | a | a | ab | b |
|  |  | *Spirochaetes* | a | a | a | a | ab | a | a | a | b |
|  |  | TM7-3 | a | a | a | a | a | a | a | a | a |
|  |  | *Erysipelotrichi* | a | a | a | a | a | a | a | a | a |
|  |  | *Mollicutes* | ab | b | a | a | a | a | a | a | ab |
|  | Order | *Bacteroidales* | bc | bc | bc | ab | c | c | bc | bc | a |
|  |  | *Fibrobacterales* | abc | cd | abc | a | abc | abc | bcd | ab | d |
|  |  | *Clostridiales* | ab | a | ab | b | a | a | a | ab | b |
|  |  | *Spirochaetales* | a | a | a | a | ab | a | a | a | b |
|  |  | CW040 | a | a | a | a | a | a | a | a | a |
|  |  | *Erysipelotrichales* | a | a | a | a | a | a | a | a | a |
|  |  | RF39 | ab | b | a | a | a | a | a | a | ab |
|  | Family | *Bacteroidales,* unknown family affiliations | ab | ab | ab | a | ab | ab | b | ab | ab |
|  |  | *Bacteroidaceae* | a | a | a | a | a | a | a | a | a |
|  |  | *Porphyromonadaceae* | a | b | a | a | ab | ab | b | a | b |
|  |  | *Prevotellaceae* | b | b | bc | b | bc | c | b | bc | a |
|  |  | *Fibrobacteraceae* | abc | cd | abc | a | abc | abc | bcd | ab | d |
|  |  | *Clostridiales,* unknown family affiliations | cde | abcd | bcd | abcd | abc | ab | a | de | e |
|  |  | *Catabacteriaceae* | a | a | a | a | a | a | a | a | a |
|  |  | *Lachnospiraceae* | abc | abc | bcd | d | ab | ab | a | abc | cd |
|  |  | *Ruminococcaceae* | a | a | a | a | a | a | a | a | a |
|  |  | *Veillonellaceae* | a | a | a | a | a | a | a | a | a |
|  |  | *Spirochaetaceae*^a^ | a | a | a | a | ab | a | a | a | b |
|  |  | F16^a^ | a | a | a | a | a | a | a | a | a |
|  |  | *Erysipelotrichaceae* | a | a | a | a | a | a | a | a | a |
|  |  | RF39, unknown family affiliations | ab | b | a | a | a | a | a | a | ab |
|  | Genus | *Bacteroidales*, unknown family and genus affiliations | ab | ab | ab | a | ab | ab | b | ab | ab |
|  |  | *Bacteroides* | a | a | a | a | a | a | a | a | a |
|  |  | *Parabacteroides* | a | a | a | a | a | a | a | a | a |
|  |  | *Prevotellaceae*, unknown genus affiliations | a | a | a | a | a | a | a | a | a |
|  |  | *Prevotella* | b | b | bc | b | bc | c | b | bc | a |
|  |  | *Fibrobacter* | abc | cd | abc | a | abc | abc | bcd | ab | d |
|  |  | *Clostridiales*, unknown family and genus affiliations | cde | abcd | bcd | abcd | abc | ab | a | de | e |
|  |  | Catabacteriaceae, unknown genus affiliations | a | a | a | a | a | a | a | a | a |
|  |  | *Lachnospiraceae*, unknown genus affiliations | abc | abc | abc | bc | ab | abc | a | ab | c |
|  |  | *Butyrivibrio* | abc | ab | cd | d | ab | a | ab | bc | abc |
|  |  | *Coprococcus* | a | a | a | a | a | a | a | a | a |
|  |  | *Pseudobutyrivibrio* | a | a | a | a | a | a | a | a | a |
|  |  | *Roseburia* | a | a | a | a | a | a | a | a | a |
|  |  | *Ruminococcaceae*, unknown genus affiliations | a | a | a | a | a | a | a | a | a |
|  |  | *Ruminococcus* | a | a | a | a | a | a | a | a | a |
|  |  | *Veillonellaceae*, unknown genus affiliations | a | a | a | a | a | a | a | a | a |
|  |  | *Treponema* | a | a | a | a | ab | a | a | a | b |
|  |  | F16, unknown genus affiliations | a | a | a | a | a | a | a | a | a |
|  |  | *Bulleidia* | a | a | a | a | a | a | a | a | a |
|  |  | *Sharpea* | a | a | a | a | a | a | a | a | a |
|  |  | RF39, unknown family and genus affiliations | ab | b | a | a | a | a | a | a | ab |
| Archaea | Mixed | *Methanobrevibacter gottschalkii* clade | ab | ab | ab | a | ab | ab | b | ab | ab |
|  |  | *Methanobrevibacter ruminantium* clade | a | a | a | a | a | a | a | a | a |
|  |  | *Methanosphaera* spp. | a | a | a | a | a | a | a | a | a |
|  |  | ‘*Methanoplasmatales*’ | a | a | a | a | a | a | a | a | a |
| Ciliate protozoa | Genus | *Entodinium* | b | b | bc | b | bc | c | b | bc | a |
|  |  | *Epidinium* | abc | cd | abc | a | abc | abc | bcd | ab | d |
|  |  | *Eremoplastron-Diploplastron* | cde | abcd | bcd | abcd | abc | ab | a | de | e |
|  |  | *Ostracodinium* | a | a | a | a | a | a | a | a | a |
|  |  | *Polyplastron* | abc | abc | abc | bc | ab | abc | a | ab | c |
|  |  | *Dasytricha* | abc | ab | cd | d | ab | a | ab | bc | abc |
|  |  | *Isotricha* 2 | a | a | a | a | a | a | a | a | a |
| Fungi | Sub-genus | *Caecomyces* 1 | a | a | a | a | a | a | a | a | a |
|  |  | KF1 | a | a | a | a | a | a | a | a | a |
|  |  | *Neocallimastix* 1 | a | a | a | a | a | a | a | a | a |
|  |  | *Orpinomyces* 5 | a | a | a | a | a | a | a | a | a |
|  |  | *Orpinomyces* 6 | a | a | a | a | a | a | a | a | a |
|  |  | *Piromyces* 2 | a | a | a | a | ab | a | a | a | b |
|  |  | *Piromyces* 7 | a | a | a | a | a | a | a | a | a |
|  |  | SK3 | a | a | a | a | a | a | a | a | a |

**B. Pasture-fed sheep**

| Microbial group | Taxonomic rank | Taxon | DNA extraction method | | | | | | | | |
| --- | --- | --- | --- | --- | --- | --- | --- | --- | --- | --- | --- |
|  |  |  | PCBB | PCFI | PCQI | PCSA | PSP1 | PSP2 | QIAG | RBBC | ZYMO |
| Bacteria | Phylum | *Bacteroidetes* | bc | cd | bc | ab | e | e | de | bc | a |
|  |  | *Fibrobacteres* | ab | c | a | a | ab | ab | bc | a | d |
|  |  | *Firmicutes* | c | bc | bc | c | a | a | ab | bc | c |
|  |  | *Spirochaetes* | ab | ab | a | a | ab | ab | ab | ab | b |
|  |  | TM7 | a | a | a | a | a | a | a | a | a |
|  |  | *Tenericutes* | b | a | bc | d | a | a | a | cd | b |
|  | Class | *Bacteroidia* | bc | cd | bc | ab | e | e | de | bc | a |
|  |  | *Fibrobacteres* | ab | c | a | a | ab | ab | bc | a | d |
|  |  | *Clostridia* | c | bc | bc | c | a | a | ab | bc | c |
|  |  | *Spirochaetes* | ab | ab | a | a | ab | ab | ab | ab | b |
|  |  | TM7-3 | a | a | a | a | a | a | a | a | a |
|  |  | *Erysipelotrichi* | b | a | bc | d | a | a | a | c | b |
|  |  | *Mollicutes* | a | a | a | a | a | a | a | a | a |
|  | Order | *Bacteroidales* | bc | cd | bc | ab | e | e | de | bc | a |
|  |  | *Fibrobacterales* | ab | c | a | a | ab | ab | bc | a | d |
|  |  | *Clostridiales* | c | bc | bc | c | a | a | ab | bc | c |
|  |  | *Spirochaetales* | ab | ab | a | a | ab | ab | ab | ab | b |
|  |  | CW040 | a | a | a | a | a | a | a | a | a |
|  |  | *Erysipelotrichales* | b | a | bc | d | a | a | a | c | b |
|  |  | RF39 | a | a | a | a | a | a | a | a | a |
|  | Family | *Bacteroidales,* unknown family affiliations | ab | a | ab | ab | b | ab | ab | ab | ab |
|  |  | *Bacteroidaceae* | ab | bc | ab | a | abc | abc | abc | ab | c |
|  |  | *Porphyromonadaceae* | abc | bc | a | a | ab | ab | ab | a | c |
|  |  | *Prevotellaceae* | bc | bcd | abc | ab | d | d | cd | bc | a |
|  |  | *Fibrobacteraceae* | ab | c | a | a | ab | ab | bc | a | d |
|  |  | *Clostridiales,* unknown family affiliations | bcd | cd | abc | abcd | ab | a | ab | abc | d |
|  |  | *Catabacteriaceae* | a | a | a | a | a | a | a | a | a |
|  |  | *Lachnospiraceae* | bcd | abcd | cd | d | a | ab | abc | abcd | d |
|  |  | *Ruminococcaceae* | bc | abc | abc | bc | ab | a | abc | bc | c |
|  |  | *Veillonellaceae* | a | a | a | a | a | a | a | a | a |
|  |  | *Spirochaetaceae*^a^ | ab | ab | a | a | ab | ab | ab | ab | b |
|  |  | F16^a^ | a | a | a | a | a | a | a | a | a |
|  |  | *Erysipelotrichaceae* | c | ab | cd | e | a | a | a | d | bc |
|  |  | RF39, unknown family affiliations | a | a | a | a | a | a | a | a | a |
|  | Genus | *Bacteroidales*, unknown family and genus affiliations | ab | a | ab | ab | b | ab | ab | ab | ab |
|  |  | *Bacteroides* | ab | bc | ab | a | abc | abc | abc | ab | c |
|  |  | *Parabacteroides* | abc | bc | a | a | ab | ab | ab | a | c |
|  |  | *Prevotellaceae*, unknown genus affiliations | a | a | a | a | a | a | a | a | a |
|  |  | *Prevotella* | bc | bcd | abc | ab | d | d | cd | bc | a |
|  |  | *Fibrobacter* | ab | c | a | a | ab | ab | bc | a | d |
|  |  | *Clostridiales*, unknown family and genus affiliations | bcd | cd | abc | abcd | ab | a | ab | abc | d |
|  |  | Catabacteriaceae, unknown genus affiliations | a | a | a | a | a | a | a | a | a |
|  |  | *Lachnospiraceae*, unknown genus affiliations | ab | ab | ab | ab | a | a | ab | ab | b |
|  |  | *Butyrivibrio* | a | a | ab | b | a | a | a | ab | ab |
|  |  | *Coprococcus* | d | abcd | cd | bcd | ab | abc | a | abcd | abcd |
|  |  | *Pseudobutyrivibrio* | a | a | a | a | a | a | a | a | a |
|  |  | *Roseburia* | a | a | a | a | a | a | a | a | a |
|  |  | *Ruminococcaceae*, unknown genus affiliations | ab | ab | ab | ab | ab | a | ab | ab | b |
|  |  | *Ruminococcus* | a | a | a | a | a | a | a | a | a |
|  |  | *Veillonellaceae*, unknown genus affiliations | a | a | a | a | a | a | a | a | a |
|  |  | *Treponema* | ab | ab | a | a | ab | ab | ab | ab | b |
|  |  | F16, unknown genus affiliations | a | a | a | a | a | a | a | a | a |
|  |  | *Bulleidia* | ab | a | c | bc | a | ab | a | abc | bc |
|  |  | *Sharpea* | c | ab | cd | e | ab | ab | a | de | bc |
|  |  | RF39, unknown family and genus affiliations | a | a | a | a | a | a | a | a | a |
| Archaea | Mixed | *Methanobrevibacter gottschalkii* clade | ab | a | ab | ab | b | ab | ab | ab | ab |
|  |  | *Methanobrevibacter ruminantium* clade | ab | bc | ab | a | abc | abc | abc | ab | c |
|  |  | *Methanosphaera* spp. | abc | bc | a | a | ab | ab | ab | a | c |
|  |  | ‘*Methanoplasmatales*’ | a | a | a | a | a | a | a | a | a |
