# Supplementary material for: Effect of DNA Extraction Methods and Sampling Techniques on the Apparent Structure of Cow and Sheep Rumen Microbial Communities
Source: PLoS One. 2013 Sep 11;8(9):e74787. doi: 10.1371/journal.pone.0074787 (PMC3770609; doi:10.1371/journal.pone.0074787)
Supplement: Table S6 — Effects of bead beating methods on DNA extractions from rumen samples. Microbial community compositions (A, % of total community), specific DNA yields (B), and the mean number of sequencing reads per sample (C). DNA was extracted in duplicate (means of duplicate determinations are shown) with the PCQI DNA extraction method from rumen contents of three sheep and one cow, employing different bead beating methods and times. (DOCX) [file pone.0074787.s007.docx]

**Table S6. Effects of bead beating methods on DNA extractions from rumen samples.** Microbial community compositions (A, % of total community), specific DNA yields (B), and the mean number of sequencing reads per sample (C). DNA was extracted in duplicate (means of duplicate determinations are shown) with the PCQI DNA extraction method from rumen contents of three sheep and one cow, employing different bead beating methods and times.

**A. Microbial community compositions**

| Microbial group (and taxonomic rank) | Taxon | Animal identifier and bead-beating method^a^ | | | | | | | | | | | |
| --- | --- | --- | --- | --- | --- | --- | --- | --- | --- | --- | --- | --- | --- |
|  |  | 723 | | | | | | 5472 | | | | | |
|  |  | 1 | 2 | 3 | 4 | 5 | FP | 1 | 2 | 3 | 4 | 5 | FP |
| Bacteria (phylum) | *Bacteroidetes* | 34.0 | 33.5 | 36.4 | 30.2 | 30.5 | 26.9 | 64.5 | 64.0 | 61.2 | 62.5 | 61.9 | 58.1 |
|  | *Fibrobacteres* | 8.2 | 7.1 | 7.4 | 5.1 | 5.6 | 7.3 | 4.8 | 3.2 | 3.1 | 3.0 | 2.5 | 2.3 |
|  | *Firmicutes* | 50.0 | 51.9 | 49.2 | 58.0 | 57.5 | 60.2 | 27.4 | 27.9 | 29.7 | 28.3 | 28.0 | 32.5 |
|  | SR1 | 1.2 | 1.0 | 0.6 | 0.9 | 1.2 | 0.4 | 0.0 | 0.0 | 0.0 | 0.0 | 0.0 | 0.0 |
|  | *Tenericutes* | 3.7 | 3.8 | 3.0 | 3.2 | 2.9 | 2.7 | 2.2 | 4.0 | 5.2 | 5.8 | 6.5 | 6.0 |
| Archaea (mixed) | *Mbb. gottschalkii* | 29.0 | 25.2 | 24.0 | 26.7 | 22.6 | 31.3 | 9.9 | 9.6 | 8.4 | 9.6 | 12.1 | 10.4 |
|  | *Mbb. ruminantium* | 52.4 | 54.8 | 60.7 | 59.8 | 63.0 | 56.2 | 42.6 | 47.3 | 49.8 | 45.6 | 41.7 | 44.9 |
|  | *Methanosphaera* | 3.7 | 3.6 | 4.5 | 3.9 | 3.0 | 5.7 | 31.4 | 33.9 | 31.4 | 36.8 | 37.0 | 41.3 |
|  | ‘*Methanoplasmatales*’ | 14.9 | 16.4 | 10.8 | 9.6 | 11.5 | 6.8 | 15.8 | 9.2 | 10.5 | 7.9 | 9.2 | 3.4 |
| Ciliate-protozoa (genus) | *Anoplodinium-Diplodinium* 1 | 0.2 | 0.3 | 0.2 | 0.0 | 0.9 | 0.9 | 5.4 | 10.7 | 9.1 | 0.8 | 8.0 | 17.2 |
|  | *Anoplodinium-Diplodinium* 2 | 0.7 | 0.0 | 0.2 | 0.0 | 0.0 | 0.0 | 2.2 | 3.9 | 0.6 | 1.1 | 1.7 | 3.1 |
|  | *Entodinium* | 34.3 | 28.9 | 31.1 | 39.1 | 36.4 | 45.9 | 9.1 | 18.1 | 17.6 | 59.8 | 17.6 | 15.2 |
|  | *Epidinium* | 0.4 | 1.9 | 0.7 | 1.0 | 2.2 | 3.1 | 17.7 | 20.4 | 23.6 | 11.0 | 22.2 | 27.2 |
|  | *Eremoplastron-Diploplastron* | 3.1 | 4.6 | 4.4 | 4.0 | 3.1 | 4.7 | 0.0 | 0.0 | 0.0 | 0.0 | 1.0 | 0.0 |
|  | *Eudiplodinium* | 0.4 | 0.5 | 0.2 | 0.0 | 0.0 | 0.2 | 1.1 | 3.2 | 5.9 | 3.0 | 10.5 | 4.9 |
|  | *Metadinium* | 0.9 | 1.6 | 1.1 | 0.0 | 0.2 | 0.0 | 0.0 | 0.0 | 0.0 | 0.0 | 0.0 | 0.0 |
|  | *Ophryoscolex* | 0.4 | 0.0 | 0.0 | 0.0 | 0.0 | 0.0 | 0.0 | 0.0 | 0.0 | 0.0 | 0.0 | 0.0 |
|  | *Ostracodinium* 1 | 0.0 | 0.0 | 0.0 | 0.2 | 0.0 | 0.0 | 0.0 | 0.0 | 0.0 | 0.0 | 0.0 | 0.0 |
|  | *Ostracodinium* 2 | 54.9 | 54.2 | 56.0 | 49.2 | 49.6 | 36.8 | 0.0 | 1.3 | 0.0 | 1.5 | 1.4 | 0.0 |
|  | *Polyplastron* | 3.3 | 2.7 | 2.0 | 3.7 | 3.2 | 3.5 | 0.0 | 0.0 | 0.0 | 0.0 | 0.0 | 0.0 |
|  | *Dasytricha* | 0.5 | 1.3 | 1.5 | 1.0 | 1.3 | 2.4 | 64.5 | 41.7 | 42.8 | 22.7 | 35.2 | 31.8 |
|  | *Isotricha* 2 | 1.1 | 4.0 | 2.5 | 2.0 | 3.1 | 2.5 | 0.0 | 0.4 | 0.0 | 0.0 | 0.0 | 0.0 |
|  | *Isotricha* 3 | 0.0 | 0.0 | 0.0 | 0.0 | 0.0 | 0.0 | 0.0 | 0.4 | 0.4 | 0.0 | 2.4 | 0.6 |
|  |  | Animal identifier and bead-beating method^a^ | | | | | | | | | | | |
|  |  | 322 | | | | | | 325 | | | | | |
|  |  | 1 | 2 | 3 | 4 | 5 | FP | 1 | 2 | 3 | 4 | 5 | FP |
| Bacteria (phylum) | *Bacteroidetes* | 63.7 | 58.1 | 50.8 | 51.7 | 47.2 | 49.1 | 60.4 | 56.2 | 62.6 | 49.7 | 50.4 | 50.4 |
|  | *Fibrobacteres* | 0.7 | 0.6 | 0.7 | 0.4 | 0.5 | 0.3 | 0.5 | 0.4 | 0.7 | 0.8 | 0.6 | 0.6 |
|  | *Firmicutes* | 31.2 | 36.8 | 44.9 | 44.6 | 48.3 | 47.5 | 35.4 | 39.9 | 32.6 | 45.2 | 45.3 | 45.7 |
|  | SR1 | 0.2 | 0.1 | 0.2 | 0.0 | 0.2 | 0.1 | 0.0 | 0.0 | 0.0 | 0.1 | 0.1 | 0.0 |
|  | *Tenericutes* | 1.8 | 1.7 | 1.5 | 1.5 | 1.5 | 1.3 | 1.6 | 1.9 | 2.1 | 2.3 | 1.4 | 1.5 |
| Archaea (mixed) | *Mbb. gottschalkii* | 23.6 | 25.6 | 39.6 | 34.0 | 37.7 | 36.3 | 30.1 | 30.1 | 27.4 | 31.5 | 31.4 | 30.6 |
|  | *Mbb. ruminantium* | 22.4 | 19.0 | 21.1 | 19.8 | 19.7 | 23.7 | 21.2 | 23.0 | 26.1 | 19.2 | 19.8 | 27.8 |
|  | *Methanosphaera* | 42.1 | 40.4 | 32.1 | 39.1 | 34.5 | 33.7 | 32.5 | 35.1 | 32.3 | 41.1 | 38.7 | 33.7 |
|  | ‘*Methanoplasmatales*’ | 11.7 | 14.8 | 7.2 | 6.9 | 7.9 | 6.3 | 16.2 | 11.7 | 14.3 | 8.1 | 10.0 | 8.0 |
| Ciliate-protozoa (genus) | *Anoplodinium-Diplodinium* 1 | 0.0 | 0.0 | 0.0 | 0.0 | 0.2 | 0.0 | 0.0 | 0.0 | 0.0 | 0.1 | 0.3 | 0.0 |
|  | *Anoplodinium-Diplodinium* 2 | 0.0 | 0.0 | 0.0 | 0.0 | 0.0 | 0.0 | 0.0 | 0.0 | 0.0 | 0.0 | 0.0 | 0.0 |
|  | *Entodinium* | 26.5 | 20.3 | 21.6 | 27.3 | 22.3 | 25.1 | 24.7 | 27.6 | 25.7 | 27.8 | 25.3 | 22.3 |
|  | *Epidinium* | 20.3 | 24.4 | 22.4 | 17.7 | 21.4 | 17.6 | 44.8 | 39.5 | 40.8 | 44.9 | 43.2 | 45.5 |
|  | *Eremoplastron-Diploplastron* | 0.0 | 0.0 | 0.0 | 0.0 | 0.0 | 0.0 | 0.0 | 0.0 | 0.0 | 0.0 | 0.0 | 0.0 |
|  | *Eudiplodinium* | 32.6 | 34.7 | 37.2 | 38.9 | 40.2 | 39.3 | 14.4 | 13.5 | 11.4 | 12.5 | 15.4 | 15.8 |
|  | *Metadinium* | 0.0 | 0.0 | 0.0 | 0.0 | 0.0 | 0.0 | 0.0 | 0.0 | 0.0 | 0.0 | 0.0 | 0.0 |
|  | *Ophryoscolex* | 0.0 | 0.0 | 0.0 | 0.0 | 0.0 | 0.0 | 0.0 | 0.0 | 0.0 | 0.0 | 0.0 | 0.0 |
|  | *Ostracodinium* 1 | 0.0 | 0.0 | 0.0 | 0.0 | 0.0 | 0.0 | 0.0 | 0.0 | 0.0 | 0.0 | 0.0 | 0.0 |
|  | *Ostracodinium* 2 | 0.0 | 0.0 | 0.0 | 0.0 | 0.0 | 0.0 | 0.0 | 0.0 | 0.0 | 0.0 | 0.0 | 0.0 |
|  | *Polyplastron* | 0.0 | 0.0 | 0.0 | 0.0 | 0.0 | 0.0 | 0.0 | 0.0 | 0.0 | 0.0 | 0.0 | 0.2 |
|  | *Dasytricha* | 17.3 | 17.2 | 14.2 | 13.4 | 12.6 | 14.9 | 2.4 | 2.1 | 1.6 | 1.2 | 1.5 | 1.6 |
|  | *Isotricha* 2 | 0.0 | 0.0 | 0.0 | 0.0 | 0.2 | 0.0 | 13.7 | 17.1 | 20.5 | 13.4 | 14.2 | 14.6 |
|  | *Isotricha* 3 | 3.3 | 3.4 | 4.6 | 2.8 | 3.2 | 3.0 | 0.0 | 0.2 | 0.0 | 0.2 | 0.0 | 0.0 |

^a^1 to 5 indicate the number of minutes samples were homogenised in the Mini-Beadbeater-96 (Biospec Products), whereas the designation FP means that samples were homogenised for 45 s at 6.5 m s^‑1^ in a FastPrep FP120 device (MP Biomedicals).

**B. Mean specific DNA yields**

| Animal identifier | 723 | | | | | | 5472 | | | | | |
| --- | --- | --- | --- | --- | --- | --- | --- | --- | --- | --- | --- | --- |
| Bead beating method^a^ | 1 | 2 | 3 | 4 | 5 | FP | 1 | 2 | 3 | 4 | 5 | FP |
| Specific DNA yield (mg g^‑1^ dry weight rumen content) | 410 | 377 | 419 | 523 | 468 | 429 | 485 | 470 | 516 | 703 | 685 | 655 |
| Animal identifier | 322 | | | | | | 325 | | | | | |
| Bead beating method^a^ | 1 | 2 | 3 | 4 | 5 | FP | 1 | 2 | 3 | 4 | 5 | FP |
| Specific DNA yield (mg g^‑1^ dry weight rumen content) | 442 | 446 | 516 | 684 | 701 | 679 | 477 | 457 | 397 | 617 | 613 | 575 |

^a^1 to 5 indicate the number of minutes samples were homogenised in the Mini-Beadbeater-96 (Biospec Products), whereas the designation FP means that samples were homogenised for 45 s at 6.5 m s^‑1^ in a FastPrep FP120 device (MP Biomedicals).

**C. Mean number of sequencing reads**

| Animal identifier | 723 | | | | | | 5472 | | | | | |
| --- | --- | --- | --- | --- | --- | --- | --- | --- | --- | --- | --- | --- |
| Bead beating method^a^ | 1 | 2 | 3 | 4 | 5 | FP | 1 | 2 | 3 | 4 | 5 | FP |
| Bacteria | 906 | 1029 | 1053 | 982 | 1451 | 1017 | 1347 | 1287 | 1770 | 2725 | 1653 | 1329 |
| Archaea | 220 | 193 | 233 | 210 | 235 | 261 | 231 | 202 | 232 | 244 | 228 | 257 |
| Ciliate protozoa | 176 | 187 | 221 | 199 | 226 | 258 | 48 | 105 | 100 | 67 | 137 | 82 |
| Animal identifier | 322 | | | | | | 325 | | | | | |
| Bead beating method^a^ | 1 | 2 | 3 | 4 | 5 | FP | 1 | 2 | 3 | 4 | 5 | FP |
| Bacteria | 1493 | 1818 | 1485 | 1234 | 1518 | 1835 | 1681 | 1378 | 1313 | 1374 | 1182 | 1174 |
| Archaea | 258 | 247 | 281 | 238 | 224 | 261 | 226 | 277 | 230 | 247 | 244 | 256 |
| Ciliate protozoa | 228 | 266 | 286 | 269 | 314 | 387 | 348 | 241 | 279 | 383 | 317 | 268 |

^a^1 to 5 indicate the number of minutes samples were homogenised in the Mini-Beadbeater-96 (Biospec Products), whereas the designation FP means that samples were homogenised for 45 s at 6.5 m s^‑1^ in a FastPrep FP120 device (MP Biomedicals).
