# Supplementary material for: Effect of DNA Extraction Methods and Sampling Techniques on the Apparent Structure of Cow and Sheep Rumen Microbial Communities
Source: PLoS One. 2013 Sep 11;8(9):e74787. doi: 10.1371/journal.pone.0074787 (PMC3770609; doi:10.1371/journal.pone.0074787)
Supplement: Table S7 — Effect of rumen sampling method on the apparent microbial community structure. Microbial community compositions (% of total community) from DNA extracted from rumen samples obtained by oral stomach tubing and through a fistula from 14 dairy cows. (DOCX) [file pone.0074787.s008.docx]

**Table S7. Effect of rumen sampling method on the apparent microbial community structure.**

Microbial community compositions (% of total community) from DNA extracted from rumen samples obtained by oral stomach tubing and through a fistula from 14 dairy cows.

| Microbial group | Taxonomic rank | Taxon | Rumen sampling method | | | | | | |
| --- | --- | --- | --- | --- | --- | --- | --- | --- | --- |
|  |  |  | Oral stomach tubing | | Fistula | | Both methods | | Significance of difference |
|  |  |  | Mean | SE^a^ | Mean | SE | Mean | SE | *p*^b^ |
| Bacteria | Phylum | *Actinobacteria* | 1.6 | 0.2 | 1.7 | 0.2 | 1.6 | 0.2 | 0.797 |
|  |  | *Bacteroidetes* | 45.5 | 2.5 | 40.5 | 2.0 | 43.0 | 2.3 | 0.050 |
|  |  | *Fibrobacteres* | 2.3 | 0.6 | 2.1 | 0.5 | 2.2 | 0.5 | 0.689 |
|  |  | *Firmicutes* | 33.2 | 3.5 | 38.3 | 3.9 | 35.7 | 3.7 | 0.017 |
|  |  | *Tenericutes* | 15.9 | 4.8 | 15.5 | 4.7 | 15.7 | 4.7 | 0.790 |
|  | Class | *Actinobacteria*^c^ | 1.6 | 0.2 | 1.7 | 0.2 | 1.6 | 0.2 | 0.797 |
|  |  | *Bacteroidia* | 45.4 | 2.5 | 40.5 | 2.0 | 43.0 | 2.3 | 0.051 |
|  |  | *Fibrobacteres* | 2.3 | 0.6 | 2.1 | 0.5 | 2.2 | 0.5 | 0.689 |
|  |  | *Bacilli* | 2.6 | 1.2 | 1.1 | 0.4 | 1.8 | 0.9 | 0.075 |
|  |  | *Clostridia* | 30.6 | 4.0 | 37.2 | 4.2 | 33.9 | 4.1 | 0.001 |
|  |  | *Erysipelotrichi* | 15.3 | 4.9 | 15.0 | 4.7 | 15.1 | 4.7 | 0.847 |
|  | Order | *Coriobacteriales* | 1.3 | 0.2 | 1.4 | 0.2 | 1.4 | 0.2 | 0.560 |
|  |  | *Bacteroidales*^c^ | 45.4 | 2.5 | 40.5 | 2.0 | 43.0 | 2.3 | 0.051 |
|  |  | *Fibrobacterales*^c^ | 2.3 | 0.6 | 2.1 | 0.5 | 2.2 | 0.5 | 0.689 |
|  |  | *Lactobacillales*^c^ | 2.6 | 1.2 | 1.0 | 0.4 | 1.8 | 0.9 | 0.075 |
|  |  | *Clostridiales*^c^ | 30.6 | 4.0 | 37.2 | 4.2 | 33.9 | 4.1 | 0.001 |
|  |  | *Erysipelotrichales*^c^ | 15.3 | 4.9 | 15.0 | 4.7 | 15.1 | 4.7 | 0.847 |
|  | Family | *Coriobacteriaceae* | 1.2 | 0.2 | 1.4 | 0.2 | 1.3 | 0.2 | 0.536 |
|  |  | *Bacteroidales*, unknown family affiliations | 13.5 | 1.6 | 15.4 | 1.4 | 14.4 | 1.5 | 0.141 |
|  |  | *Prevotellaceae* | 31.3 | 2.6 | 24.8 | 1.8 | 28.0 | 2.4 | 0.006 |
|  |  | *Fibrobacteraceae*^c^ | 2.3 | 0.6 | 2.1 | 0.5 | 2.2 | 0.5 | 0.689 |
|  |  | *Streptococcaceae* | 2.5 | 1.2 | 1.0 | 0.4 | 1.8 | 0.9 | 0.078 |
|  |  | *Clostridiales*, unknown family affiliations | 6.1 | 1.3 | 6.5 | 1.3 | 6.3 | 1.3 | 0.286 |
|  |  | *Clostridiales* family XIII *Incertae Sedis* | 1.3 | 0.1 | 1.9 | 0.2 | 1.6 | 0.2 | 0.036 |
|  |  | *Lachnospiraceae* | 14.5 | 2.0 | 20.1 | 2.2 | 17.3 | 2.2 | 0.001 |
|  |  | *Ruminococcaceae* | 6.0 | 1.0 | 6.2 | 0.9 | 6.1 | 0.9 | 0.451 |
|  |  | *Veillonellaceae* | 2.3 | 0.9 | 2.0 | 0.6 | 2.2 | 0.7 | 0.420 |
|  |  | *Erysipelotrichaceae* | 15.0 | 4.9 | 14.9 | 4.7 | 15.0 | 4.7 | 0.938 |
|  | Genus | Bacteroidales, unknown family and genus affiliations^c^ | 13.5 | 1.6 | 15.4 | 1.4 | 14.4 | 1.5 | 0.141 |
|  |  | *Prevotellaceae,* unknown genus affiliations | 2.0 | 0.3 | 1.4 | 0.1 | 1.7 | 0.2 | 0.012 |
|  |  | *Prevotella* | 29.2 | 2.6 | 23.4 | 1.8 | 26.3 | 2.3 | 0.008 |
|  |  | *Fibrobacter*^c^ | 2.3 | 0.6 | 2.1 | 0.5 | 2.2 | 0.5 | 0.689 |
|  |  | *Streptococcus*^c^ | 2.5 | 1.2 | 1.0 | 0.4 | 1.8 | 0.9 | 0.078 |
|  |  | *Clostridiales*, unknown family and genus affiliations^c^ | 6.1 | 1.3 | 6.5 | 1.3 | 6.3 | 1.3 | 0.286 |
|  |  | *Clostridiales* family XIII *Incertae Sedis*, unknown genus affiliations | 1.1 | 0.1 | 1.7 | 0.2 | 1.4 | 0.2 | 0.020 |
|  |  | *Lachnospiraceae*, unknown genus affiliations | 6.1 | 0.9 | 8.4 | 1.0 | 7.2 | 1.0 | 0.000 |
|  |  | *Butyrivibrio* | 5.2 | 1.0 | 6.6 | 1.0 | 5.9 | 1.0 | 0.039 |
|  |  | *Coprococcus* | 1.7 | 0.2 | 2.5 | 0.2 | 2.1 | 0.2 | 0.001 |
|  |  | *Ruminococcaceae*, unknown genus affiliations | 5.1 | 0.8 | 5.1 | 0.7 | 5.1 | 0.8 | 0.969 |
|  |  | *Megasphaera* | 1.2 | 0.9 | 0.6 | 0.5 | 0.9 | 0.7 | 0.240 |
|  |  | *Bulleidia* | 0.8 | 0.1 | 1.0 | 0.1 | 0.9 | 0.1 | 0.050 |
|  |  | *Sharpea* | 14.1 | 4.9 | 13.7 | 4.7 | 13.9 | 4.7 | 0.805 |
| Archaea | Mixed taxonomic ranks | *Methanobrevibacter gottschalkii* clade | 41.5 | 2.8 | 39.8 | 3.0 | 40.6 | 2.0 | 0.178 |
|  |  | *Methanobrevibacter ruminantium* clade | 38.9 | 2.8 | 46.3 | 3.1 | 42.6 | 2.2 | 0.000 |
|  |  | *Methanosphaera* spp. | 10.0 | 0.8 | 8.8 | 0.6 | 9.4 | 0.5 | 0.128 |
|  |  | ‘*Methanoplasmatales*’ | 9.6 | 1.4 | 5.1 | 0.7 | 7.4 | 0.9 | 0.002 |
| Ciliate protozoa | Genus | *Anoplodinium-Diplodinium* | 2.3 | 0.7 | 1.5 | 0.4 | 1.9 | 0.4 | 0.118 |
|  |  | *Entodinium* | 6.0 | 1.0 | 2.9 | 0.4 | 4.4 | 0.6 | 0.002 |
|  |  | *Epidinium* | 56.2 | 3.9 | 67.8 | 3.6 | 62.0 | 2.8 | 0.000 |
|  |  | *Eremoplastron-Diploplastron* | 5.4 | 0.9 | 2.3 | 0.5 | 3.8 | 0.6 | 0.000 |
|  |  | *Eudiplodinium* | 8.3 | 0.9 | 5.7 | 0.8 | 7.0 | 0.6 | 0.005 |
|  |  | *Ostracodinium* | 12.4 | 1.9 | 11.1 | 1.9 | 11.7 | 1.3 | 0.219 |
|  |  | *Dasytricha* | 7.7 | 1.6 | 6.9 | 1.6 | 7.3 | 1.1 | 0.530 |
| Fungi | Sub-genus | *Anaeromyces* 1 | 3.3 | 1.4 | 4.0 | 1.7 | 3.6 | 1.1 | 0.174 |
|  |  | *Caecomyces* 1 | 36.4 | 8.0 | 30.4 | 7.2 | 33.4 | 5.3 | 0.018 |
|  |  | *Cyllamyces* 2 | 21.0 | 7.6 | 20.6 | 7.6 | 20.8 | 5.3 | 0.552 |
|  |  | *Neocallimastix* 1 | 6.2 | 1.6 | 8.4 | 2.1 | 7.3 | 1.3 | 0.015 |
|  |  | *Orpinomyces* 1 | 4.1 | 1.8 | 5.2 | 2.2 | 4.6 | 1.4 | 0.067 |
|  |  | *Piromyces* 2 | 3.8 | 1.4 | 5.0 | 1.6 | 4.4 | 1.0 | 0.011 |
|  |  | *Piromyces* 3 | 5.1 | 2.3 | 6.1 | 2.5 | 5.6 | 1.7 | 0.002 |
|  |  | *Piromyces* 6 | 1.3 | 0.6 | 1.4 | 0.5 | 1.3 | 0.4 | 0.507 |
|  |  | *Piromyces* 7 | 5.4 | 1.8 | 8.0 | 3.0 | 6.7 | 1.7 | 0.119 |
|  |  | SK3 | 6.6 | 2.1 | 5.0 | 1.7 | 5.8 | 1.3 | 0.026 |
|  |  | No BLAST hit | 4.4 | 1.8 | 3.7 | 1.6 | 4.0 | 1.2 | 0.028 |

^a^SE, Standard error.

^b^*p*, Probability that the abundance of microbial groups is not significantly different calculated with a two-tailed, dependent sample *t*-test.

^c^Value similar to that of the next highest taxonomic rank that contains that group.
