# Supplementary material for: Effect of DNA Extraction Methods and Sampling Techniques on the Apparent Structure of Cow and Sheep Rumen Microbial Communities
Source: PLoS One. 2013 Sep 11;8(9):e74787. doi: 10.1371/journal.pone.0074787 (PMC3770609; doi:10.1371/journal.pone.0074787)
Supplement: Table S8 — Effect of rumen sample fractionation on the apparent microbial community structure. Microbial community compositions (% of total community) from rumen sample fractions (liquid, solid and total) of 16 dairy cows. (DOCX) [file pone.0074787.s009.docx]

**Table S8. Effect of rumen sample fractionation on the apparent microbial community structure.**

Microbial community compositions (% of total community) from rumen sample fractions (liquid, solid and total) of 16 dairy cows.

| Microbial group | Taxonomic rank | Taxon | Rumen sample fraction | | | | | | | | | | |
| --- | --- | --- | --- | --- | --- | --- | --- | --- | --- | --- | --- | --- | --- |
|  |  |  | Liquid (L) | | Solid (S) | | Total (T) | | All | | Significance of difference | | |
|  |  |  |  |  |  |  |  |  |  |  | *p*^b^ | | |
|  |  |  | Mean | SE^a^ | Mean | SE | Mean | SE | Mean | SE | L *v* S | L *v* T | S *v* T |
| Bacteria | Phylum | *Actinobacteria* | 1.4 | 0.2 | 1.2 | 0.1 | 1.6 | 0.2 | 1.4 | 0.1 | 0.479 | 0.235 | 0.032 |
|  |  | *Bacteroidetes* | 49.7 | 3.1 | 41.1 | 1.7 | 40.8 | 1.9 | 43.9 | 1.8 | 0.017 | 0.001 | 0.877 |
|  |  | *Fibrobacteres* | 1.8 | 0.4 | 2.2 | 0.4 | 2.1 | 0.4 | 2.0 | 0.3 | 0.112 | 0.432 | 0.454 |
|  |  | *Firmicutes* | 31.1 | 2.8 | 42.6 | 3.3 | 39.3 | 3.7 | 37.7 | 2.5 | 0.000 | 0.002 | 0.014 |
|  |  | *Tenericutes* | 14.7 | 5.0 | 11.2 | 3.1 | 14.2 | 4.5 | 13.3 | 3.0 | 0.235 | 0.796 | 0.064 |
|  | Class | *Actinobacteria*^c^ | 1.4 | 0.2 | 1.2 | 0.1 | 1.6 | 0.2 | 1.4 | 0.1 | 0.479 | 0.235 | 0.032 |
|  |  | *Bacteroidia* | 49.7 | 3.1 | 41.0 | 1.7 | 40.8 | 1.9 | 43.9 | 1.8 | 0.017 | 0.001 | 0.882 |
|  |  | *Fibrobacteres*^c^ | 1.8 | 0.4 | 2.2 | 0.4 | 2.1 | 0.4 | 2.0 | 0.3 | 0.112 | 0.432 | 0.454 |
|  |  | *Bacilli* | 2.2 | 0.9 | 0.7 | 0.3 | 1.0 | 0.4 | 1.3 | 0.4 | 0.023 | 0.029 | 0.036 |
|  |  | *Clostridia* | 28.9 | 3.1 | 41.9 | 3.5 | 38.3 | 4.0 | 36.4 | 2.7 | 0.000 | 0.000 | 0.007 |
|  |  | *Erysipelotrichi* | 13.4 | 5.1 | 10.6 | 3.2 | 13.7 | 4.5 | 12.5 | 3.0 | 0.344 | 0.867 | 0.056 |
|  |  | *Mollicutes* | 1.2 | 0.2 | 0.6 | 0.1 | 0.5 | 0.1 | 0.8 | 0.1 | 0.000 | 0.000 | 0.096 |
|  | Order | *Coriobacteriales* | 1.1 | 0.2 | 1.1 | 0.1 | 1.4 | 0.2 | 1.2 | 0.1 | 0.746 | 0.196 | 0.045 |
|  |  | *Bacteroidales*^c^ | 49.7 | 3.1 | 41.0 | 1.7 | 40.8 | 1.9 | 43.9 | 1.8 | 0.017 | 0.001 | 0.882 |
|  |  | *Fibrobacterales*^c^ | 1.8 | 0.4 | 2.2 | 0.4 | 2.1 | 0.4 | 2.0 | 0.3 | 0.112 | 0.432 | 0.454 |
|  |  | *Lactobacillales* | 2.1 | 0.9 | 0.7 | 0.3 | 1.0 | 0.4 | 1.3 | 0.4 | 0.025 | 0.031 | 0.033 |
|  |  | *Clostridiales*^c^ | 28.9 | 3.1 | 41.9 | 3.5 | 38.3 | 4.0 | 36.4 | 2.7 | 0.000 | 0.000 | 0.007 |
|  |  | *Erysipelotrichales*^c^ | 13.4 | 5.1 | 10.6 | 3.2 | 13.7 | 4.5 | 12.5 | 3.0 | 0.344 | 0.867 | 0.056 |
|  | Family | *Coriobacteriaceae* | 1.1 | 0.1 | 1.0 | 0.1 | 1.4 | 0.2 | 1.2 | 0.1 | 0.680 | 0.214 | 0.043 |
|  |  | *Bacteroidales*, unknown family affiliations | 12.7 | 1.6 | 13.4 | 1.3 | 15.3 | 1.3 | 13.8 | 1.0 | 0.489 | 0.032 | 0.034 |
|  |  | *Prevotellaceae* | 36.1 | 2.7 | 27.0 | 2.0 | 25.1 | 1.7 | 29.4 | 1.8 | 0.012 | 0.000 | 0.124 |
|  |  | *Fibrobacteraceae*^c^ | 1.8 | 0.4 | 2.2 | 0.4 | 2.1 | 0.4 | 2.0 | 0.3 | 0.112 | 0.432 | 0.454 |
|  |  | *Streptococcaceae* | 2.1 | 0.9 | 0.7 | 0.3 | 0.9 | 0.4 | 1.2 | 0.4 | 0.024 | 0.031 | 0.036 |
|  |  | *Clostridiales*, unknown family affiliations | 4.4 | 0.8 | 6.3 | 1.1 | 6.7 | 1.2 | 5.8 | 0.8 | 0.000 | 0.000 | 0.367 |
|  |  | *Clostridiales* Family XIII *Incertae Sedis* | 2.2 | 0.3 | 2.2 | 0.2 | 2.0 | 0.3 | 2.1 | 0.2 | 0.894 | 0.525 | 0.422 |
|  |  | *Lachnospiraceae* | 12.7 | 1.4 | 24.7 | 2.0 | 20.8 | 2.1 | 19.4 | 1.6 | 0.000 | 0.000 | 0.002 |
|  |  | *Ruminococcaceae* | 6.1 | 0.9 | 6.6 | 0.8 | 6.5 | 0.8 | 6.4 | 0.6 | 0.138 | 0.149 | 0.518 |
|  |  | *Veillonellaceae* | 3.2 | 1.6 | 1.6 | 0.4 | 1.9 | 0.6 | 2.2 | 0.7 | 0.213 | 0.200 | 0.372 |
|  |  | *Erysipelotrichaceae* | 12.9 | 5.1 | 10.5 | 3.2 | 13.6 | 4.5 | 12.3 | 3.0 | 0.423 | 0.695 | 0.056 |
|  | Genus | *Bacteroidales*, unknown family and genus affiliations^c^ | 12.7 | 1.6 | 13.4 | 1.3 | 15.3 | 1.3 | 13.8 | 1.0 | 0.489 | 0.032 | 0.034 |
|  |  | *Prevotellaceae*, unknown genus affiliations | 3.3 | 0.4 | 1.3 | 0.1 | 1.4 | 0.1 | 2.0 | 0.3 | 0.000 | 0.000 | 0.192 |
|  |  | *Prevotella* | 32.7 | 2.5 | 25.8 | 2.0 | 23.7 | 1.7 | 27.4 | 1.6 | 0.030 | 0.001 | 0.089 |
|  |  | *Fibrobacter*^c^ | 1.8 | 0.4 | 2.2 | 0.4 | 2.1 | 0.4 | 2.0 | 0.3 | 0.112 | 0.432 | 0.454 |
|  |  | *Streptococcus*^c^ | 2.1 | 0.9 | 0.7 | 0.3 | 0.9 | 0.4 | 1.2 | 0.4 | 0.024 | 0.031 | 0.036 |
|  |  | *Clostridiales*, unknown family and genus affiliations^c^ | 4.4 | 0.8 | 6.3 | 1.1 | 6.7 | 1.2 | 5.8 | 0.8 | 0.000 | 0.000 | 0.367 |
|  |  | *Clostridiales* Family XIII *Incertae Sedis*, unknown genus affiliations | 2.0 | 0.2 | 2.0 | 0.2 | 1.8 | 0.2 | 1.9 | 0.2 | 0.847 | 0.503 | 0.415 |
|  |  | *Lachnospiraceae*, unknown genus affiliations | 5.6 | 0.6 | 10.7 | 0.9 | 8.5 | 0.9 | 8.3 | 0.7 | 0.000 | 0.000 | 0.000 |
|  |  | *Butyrivibrio* | 3.9 | 0.7 | 7.7 | 1.0 | 7.2 | 1.0 | 6.2 | 0.7 | 0.000 | 0.000 | 0.242 |
|  |  | *Coprococcus* | 1.7 | 0.2 | 3.3 | 0.3 | 2.7 | 0.3 | 2.6 | 0.2 | 0.000 | 0.000 | 0.033 |
|  |  | *Pseudobutyrivibrio* | 0.4 | 0.1 | 1.1 | 0.3 | 0.6 | 0.2 | 0.7 | 0.2 | 0.003 | 0.028 | 0.002 |
|  |  | *Ruminococcaceae*, unknown genus affiliations | 5.5 | 0.9 | 5.2 | 0.6 | 5.4 | 0.7 | 5.3 | 0.5 | 0.451 | 0.739 | 0.276 |
|  |  | *Ruminococcus* | 0.4 | 0.1 | 1.2 | 0.3 | 0.8 | 0.2 | 0.8 | 0.2 | 0.001 | 0.002 | 0.014 |
|  |  | *Veillonellaceae*, unknown genus affiliations | 1.0 | 0.2 | 0.8 | 0.1 | 0.9 | 0.1 | 0.9 | 0.1 | 0.227 | 0.259 | 0.589 |
|  |  | *Megasphaera* | 1.8 | 1.6 | 0.4 | 0.2 | 0.6 | 0.4 | 0.9 | 0.7 | 0.315 | 0.278 | 0.569 |
|  |  | *Bulleidia* | 1.0 | 0.1 | 1.1 | 0.2 | 1.0 | 0.1 | 1.1 | 0.1 | 0.346 | 0.594 | 0.465 |
|  |  | *Sharpea* | 11.7 | 5.1 | 9.0 | 3.1 | 12.3 | 4.5 | 11.0 | 3.0 | 0.382 | 0.749 | 0.051 |
| Archaea | Mixed taxonomic ranks | *Methanobrevibacter gottschalkii* clade | 60.1 | 2.5 | 41.6 | 3.1 | 40.7 | 3.1 | 47.5 | 2.6 | 0.000 | 0.000 | 0.275 |
|  |  | *Methanobrevibacter ruminantium* clade | 23.8 | 2.5 | 45.3 | 3.0 | 45.7 | 3.1 | 38.2 | 2.8 | 0.000 | 0.000 | 0.565 |
|  |  | *Methanosphaera* spp. | 9.1 | 0.9 | 9.0 | 0.8 | 8.7 | 0.6 | 8.9 | 0.5 | 0.879 | 0.640 | 0.590 |
|  |  | ‘*Methanoplasmatales*’ | 6.9 | 1.1 | 4.1 | 0.5 | 4.8 | 0.7 | 5.3 | 0.6 | 0.004 | 0.017 | 0.282 |
| Ciliate protozoa | Genus | *Anoplodinium-Diplodinium* | 2.1 | 0.5 | 1.8 | 0.4 | 1.4 | 0.4 | 1.7 | 0.3 | 0.434 | 0.114 | 0.182 |
|  |  | *Enoploplastron* | 0.7 | 0.4 | 1.0 | 0.5 | 0.8 | 0.4 | 0.8 | 0.3 | 0.036 | 0.574 | 0.418 |
|  |  | *Entodinium* | 7.6 | 1.8 | 2.0 | 0.4 | 2.6 | 0.4 | 4.1 | 0.9 | 0.002 | 0.006 | 0.053 |
|  |  | *Epidinium* | 53.0 | 4.2 | 67.4 | 3.3 | 67.8 | 3.4 | 62.8 | 2.9 | 0.000 | 0.000 | 0.772 |
|  |  | *Eremoplastron-Diploplastron* | 5.8 | 0.9 | 3.5 | 0.8 | 2.3 | 0.4 | 3.9 | 0.6 | 0.002 | 0.000 | 0.028 |
|  |  | *Eudiplodinium* | 5.6 | 0.8 | 5.8 | 1.0 | 5.6 | 0.7 | 5.7 | 0.6 | 0.876 | 0.950 | 0.750 |
|  |  | *Ostracodinium* | 13.1 | 1.9 | 12.2 | 1.9 | 11.8 | 1.8 | 12.4 | 1.3 | 0.404 | 0.191 | 0.535 |
|  |  | *Dasytricha* | 11.0 | 2.7 | 5.5 | 1.3 | 6.8 | 1.5 | 7.8 | 1.4 | 0.003 | 0.015 | 0.004 |
| Fungi | Sub-genus | *Anaeromyces* 1 | 6.4 | 3.0 | 8.1 | 4.5 | 7.3 | 4.2 | 7.3 | 2.7 | 0.373 | 0.596 | 0.098 |
|  |  | *Caecomyces* 1 | 23.9 | 6.8 | 28.8 | 7.4 | 28.1 | 7.0 | 27.0 | 4.9 | 0.145 | 0.133 | 0.540 |
|  |  | *Cyllamyces* 2 | 15.4 | 6.2 | 17.1 | 6.8 | 18.8 | 7.2 | 17.1 | 4.7 | 0.087 | 0.024 | 0.020 |
|  |  | KF1 | 1.6 | 0.8 | 0.4 | 0.2 | 0.7 | 0.3 | 0.9 | 0.4 | 0.045 | 0.045 | 0.060 |
|  |  | *Neocallimastix* 1 | 4.8 | 1.5 | 6.8 | 2.1 | 7.4 | 2.1 | 6.3 | 1.3 | 0.118 | 0.060 | 0.175 |
|  |  | *Orpinomyces* 1 | 6.4 | 2.7 | 8.7 | 3.3 | 6.6 | 2.3 | 7.2 | 2.0 | 0.251 | 0.840 | 0.156 |
|  |  | *Piromyces* 2 | 5.1 | 1.8 | 4.6 | 1.8 | 4.5 | 1.6 | 4.7 | 1.2 | 0.473 | 0.441 | 0.728 |
|  |  | *Piromyces* 3 | 7.1 | 3.5 | 5.1 | 2.2 | 5.4 | 2.4 | 5.8 | 1.9 | 0.190 | 0.200 | 0.268 |
|  |  | *Piromyces* 6 | 1.9 | 0.8 | 1.4 | 0.6 | 1.2 | 0.5 | 1.5 | 0.5 | 0.124 | 0.081 | 0.218 |
|  |  | *Piromyces* 7 | 6.7 | 2.2 | 6.8 | 2.7 | 7.1 | 2.9 | 6.9 | 1.8 | 0.966 | 0.814 | 0.366 |
|  |  | SK3 | 15.8 | 6.6 | 7.2 | 3.1 | 8.0 | 3.9 | 10.3 | 3.4 | 0.060 | 0.060 | 0.490 |
|  |  | No blast hit | 3.2 | 1.4 | 3.6 | 1.6 | 3.4 | 1.5 | 3.4 | 1.0 | 0.132 | 0.100 | 0.284 |

^a^SE, Standard error.

^b^*p*, Probability that the abundance of microbial groups is not significantly different calculated with a two-tailed, dependent sample *t*-test.

^c^Value similar to that of the next highest taxonomic rank that contains that group.
